# A measles virus encoding a CD19/CD3 bispecific T cell engager shows enhanced preclinical anti-BCP-ALL efficacy without significant toxicity

**DOI:** 10.1016/j.omton.2026.201127

**Published:** 2026-01-10

**Authors:** Sabine Heinze, Giovanna L. Stadler, Yonghui Zhang, Christine E. Engeland, Thomas F.E. Barth, Johannes P.W. Heidbuechel, Lüder H. Meyer, Michael D. Mühlebach, Klaus-Michael Debatin, Carmen Dorneburg, Christian Beltinger

**Affiliations:** 1 Department of Pediatrics and Adolescent Medicine, University Medical Center Ulm, 89075 Ulm, Germany; 2 Henan Key Laboratory of Stem Cell Clinical Application and Key Technology, Henan Provincial People’s Hospital, People’s Hospital of Zhengzhou University, Henan 450003, China; 3 Experimental Hematology and Immunotherapy, Medical Department for Haematology, Cell Therapy, Hemostaseology and Infectiology and Medical Faculty, Leipzig University, 04103 Leipzig, Germany; 4 Institute of Pathology, University Medical Center Ulm, 89081 Ulm, Germany; 5 Clinical Cooperation Unit Virotherapy, German Cancer Research Center (DKFZ), 69120 Heidelberg, Germany; 6 Division Veterinary Medicine, Paul-Ehrlich-Institut, 63225 Langen, Germany; 7 German Center for Child and Adolescent Health (DZKJ), partner site Ulm, 89075 Ulm, Germany

**Keywords:** MT: Regular Issue, oncolytic measles virus, Virotherapy, acute lymphoblastic leukemia, bsTE, bispecific T cell engager, toxicity

## Abstract

Children with high-risk B-cell precursor acute lymphoblastic leukemia (BCP-ALL) continue to face relapse and treatment resistance, emphasizing the urgent need for novel therapeutic strategies. In this study, we developed and characterized a recombinant oncolytic measles virus (MV-Blina) engineered to locally secrete a bispecific T cell engager (bsTE) targeting CD19 and CD3 (secBlina) to enhance antitumor immunity while minimizing systemic toxicity. MV-Blina demonstrated superior replication kinetics and cytotoxicity *in vitro* compared to the parental MV-Edm strain. MV-Blina infected ALL cells, secreted functional secBlina capable of engaging and activating T cells, leading to selective leukemia cell death. In *in vitro* and *in vivo* models, including patient-derived xenografts, MV-Blina demonstrated an additive yet heterogeneous anti-leukemic effect, with significant survival benefits and reduced CNS leukemic burden in MV-Blina-treated mice. Importantly, MV-Blina did not induce either short- or long-term toxicity in *in vitro* neuronal models encompassing PBMCs, nor in immunocompromised CD46 transgenic mice, even under additional immunosuppression. Collectively, these findings support further investigations of MV-Blina as a potential treatment for patients with relapsed or refractory BCP-ALL.

## Graphical abstract

**Figure:**
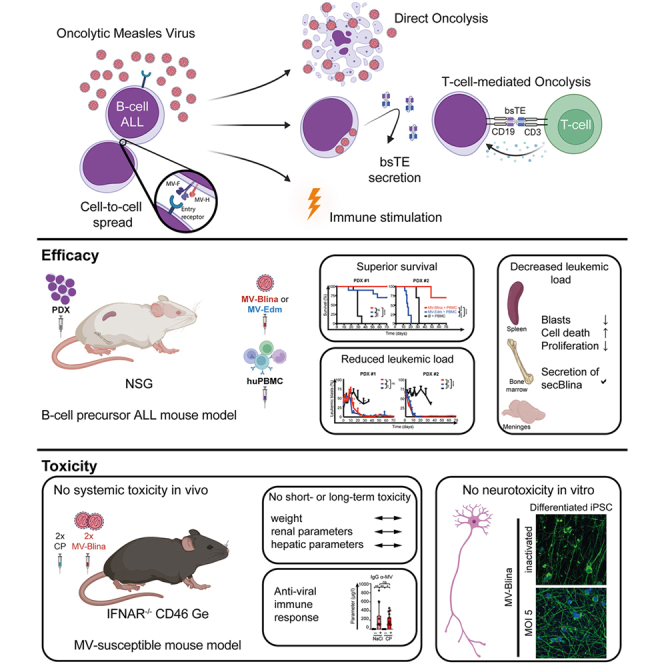

## Teaser

Beltinger and colleagues present MV-Blina, an oncolytic measles virus engineered to secrete a CD19/CD3 bispecific T cell engager with enhanced preclinical efficacy against BCP-ALL without significant toxicity. This work supports further investigations of MV-Blina as a potential treatment for patients with relapsed or refractory BCP-ALL.

## Introduction

B-cell precursor acute lymphoblastic leukemia (BCP-ALL) is the most common cancer of childhood. Although cure rates for pediatric ALL now exceed 90%, patients with high-risk (HR) ALL still face increased rates of treatment failure and relapse.^1^^,^^2^ Immunotherapeutic strategies using CAR T cells or bispecific T cell engagers (bsTEs) induce remarkable remission rates in relapsed and refractory ALL.^3^^,^^4^^,^^5^^,^^6^ Blinatumomab (Blincyto), the first bsTE to receive regulatory approval, is now being successfully employed not only in relapsed or refractory BCP-ALL,^7^^,^^8^ but also in standard-risk disease.^9^^,^^10^ This CD19/CD3-bsTE specifically binds to CD19^+^ leukemic B-cell blasts and engages T cells, thereby potentiating the killing of the leukemic blasts. However, due to the short half-life of the CD19/CD3-bsTE, patients require continuous intravenous infusion over several weeks per cycle. A subset of patients also experiences severe adverse events, in particular cytokine release syndrome and neurotoxicity.^5^^,^^6^^,^^11^^,^^12^ Similar to CD19-CAR T cell therapy, CD19/CD3-bsTE therapy is associated with the antigen evasion of leukemic blasts, by decreasing CD19 target surface expression, leading to reduced therapy efficacy.^6^ Therefore, optimizing dosing strategies and toxicity management is warranted.

Oncolytic viruses (OVs) are natural or engineered viruses that selectively target and directly kill tumor cells while activating the immune system to augment lytic cell death.^13^^,^^14^ Unlike normal cells, cancer cells have impaired virus-sensing mechanisms and defects in interferon response.^15^ Upon infection, cancer cells are eradicated by direct mechanisms, including endoplasmic reticulum stress-induced lysis and genotoxic damage from viral replication,^16^ as well as by indirect pathways, such as the activation of antitumor immune responses.^13^ Tumor-specific immunity is enhanced by the release of tumor-associated antigens (TAAs), danger- and pathogen-associated molecular patterns (DAMPs and PAMPs), and immune-stimulating cytokines and chemokines.^13^^,^^14^^,^^16^

A potent OV is the oncolytic measles virus (OMV) that infects cells via the entry receptors CD46, Nectin-4, and CD150.^17^ In addition to wild-type MV isolates, the attenuated MV Edmonston B vaccine strain (MV-Edm) also exploits CD46, that is often overexpressed on cancer cells.^16^^,^^18^^,^^19^ Subsequently, OMV preferentially replicates in and lyses cancer cells. OMVs are or have been in clinical trials for several solid tumors, demonstrating promising therapeutic potential.^20^^,^^21^^,^^22^^,^^23^^,^^24^^,^^25^^,^^26^^,^^27^^,^^28^^,^^29^^,^^30^^,^^31^^,^^32^ Notably, OMV induced a durable clinical response in a patient with refractory multiple myeloma, the first such documented case.^33^

We and others have demonstrated that OMV has preclinical efficacy in ALL.^34^^,^^35^^,^^36^^,^^37^ Specifically, we have shown that MV-Edm effectively infects, proliferates within and eradicates BCP-ALL cells *in vitro* and *in vivo*.^34^ MV-Edm caused rapid and extensive tumor cell death in ALL, in contrast to the slower cytotoxic kinetics observed in chronic lymphocytic leukemia (CLL).^36^ *In vivo*, MV-Edm treatment resulted in complete tumor regression in ALL xenograft models, highlighting its strong therapeutic potential as a replicating virus-based therapy for adult ALL.^36^ However, the effectiveness of an OMV against chemoinsensitive and relapsed ALL remains to be shown, in particular in the setting of high leukemic load and immunocompromised patients.

Wild-type MV is highly pathogenic and can induce severe pathologies, including acute encephalitis, giant cell pneumonitis, fulminant measles, and subacute sclerosing panencephalitis.^38^^,^^39^ Widespread vaccination has been effective in preventing such complications.^38^ Notably, MV vaccination has been administered intramuscularly in children, including some with ALL, with very few significant side effects. Although an early case report in 1962 of fatal atypical measles in a pediatric ALL patient raised concerns,^40^ later studies using more attenuated strains and stricter exclusion criteria demonstrated safe vaccination and effective seroconversion, leading to early measles revaccination in patients with ALL.^41^^,^^42^ While the intracranial administration of MV-Edm in mice led to encephalitis and high mortality,^43^^,^^44^ recombinant measles virus strains administered intravenously exhibited no clinical signs of toxicity, underscoring their safety.^45^^,^^46^^,^^47^

OMV toxicity studies are commonly conducted both *in vitro* and in a susceptible mouse model transgenic for the human entry receptor CD46.^44^ Furthermore, these mice are interferon type I receptor-deficient (IFNAR^−/−^) to suppress the interferon-mediated antiviral response.^44^

Combining OMVs with bsTEs localizes and amplifies T cell-mediated anti-tumor responses directly within the tumor microenvironment, thus reducing systemic toxicity and overcoming immunosuppressive barriers.^46^^,^^48^^,^^49^^,^^50^^,^^51^

This preclinical study shows that a measles virus encoding a secreted CD19/CD3-bsTE controls BCP-ALL with enhanced efficacy and without marked toxicity.

## Results

### Marked replication rate and cytotoxicity of recombinant measles virus-Blina in Vero cells

**Figure 1:**
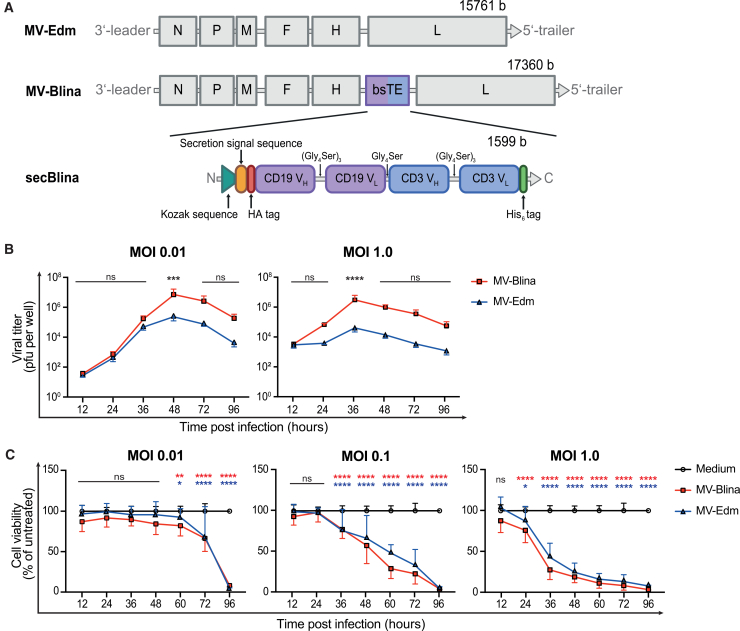
MV-Blina, an oncolytic measles virus encoding the CD19/CD3-bispecific T cell engager, replicates and is cytotoxic (A) Schematic of MV-Blina. Genomes of the parental MV-Edm strain and the recombinant MV-Blina. The bsTE construct contains the anti-human-CD19 variable heavy/light domain (CD19 V_H/L_) and the anti-human-CD3 variable heavy/light domain (CD3 V_H/L_). (B) Enhanced replication of MV-Blina compared to MV-Edm. Replication of MV-Blina was assessed by infecting Vero cells at MOI 0.01 or 1.0 with MV-Blina and MV-Edm, respectively. Viral titers at the indicated time points were determined with a titration assay. Results are shown as means ± SD of *n* = 3 from independent experiments seeded in duplicates. (C) Comparable cytotoxicity of MV-Blina and parental MV-Edm. Vero cells were infected with MOI 0.01, 0.1, or 1.0 of MV-Blina and MV-Edm, respectively. Cell viability was determined using the MTT assay at the indicated time points. Results are shown as means ± SD of *n* ≥ 3 from independent experiments seeded in sextuplicates. Statistical analysis was performed using two-way ANOVA with Dunnett’s correction. ns, not significant; ∗, *p* < 0.05; ∗∗, *p* < 0.01; ∗∗∗, *p* < 0.001; and ∗∗∗∗, *p* < 0.0001.

To develop a targeted therapy for B-cell precursor ALL, we modified the genome of MV-Edm with the sequence of a CD19/CD3-bsTE, aiming for a dual therapeutic effect: high cell kill upon MV infection as the first-line impact, followed by the local secretion of secBlina in infected but persistent blasts. This novel recombinant MV-construct, called MV-Blina, encodes a CD19/CD3-bsTE, a secretion signal, and HA- and His-tags for bsTE detection (Figure 1A). The secreted CD19/CD3-bsTE (secBlina) binds to CD3 T cells and targets the BCP-ALL cell surface marker CD19. Enhanced time- and dose-dependent replication and equivalent cytotoxicity of MV-Blina compared to parental MV-Edm was observed in Vero indicator cells, even at low multiplicities of infection (MOI) (Figures 1B and 1C). Taken together, these data demonstrate the enhanced function of MV-Blina in Vero cells.

### Acute lymphoblastic leukemia cells infected with measles virus-Blina secrete functional secBlina

**Figure 2:**
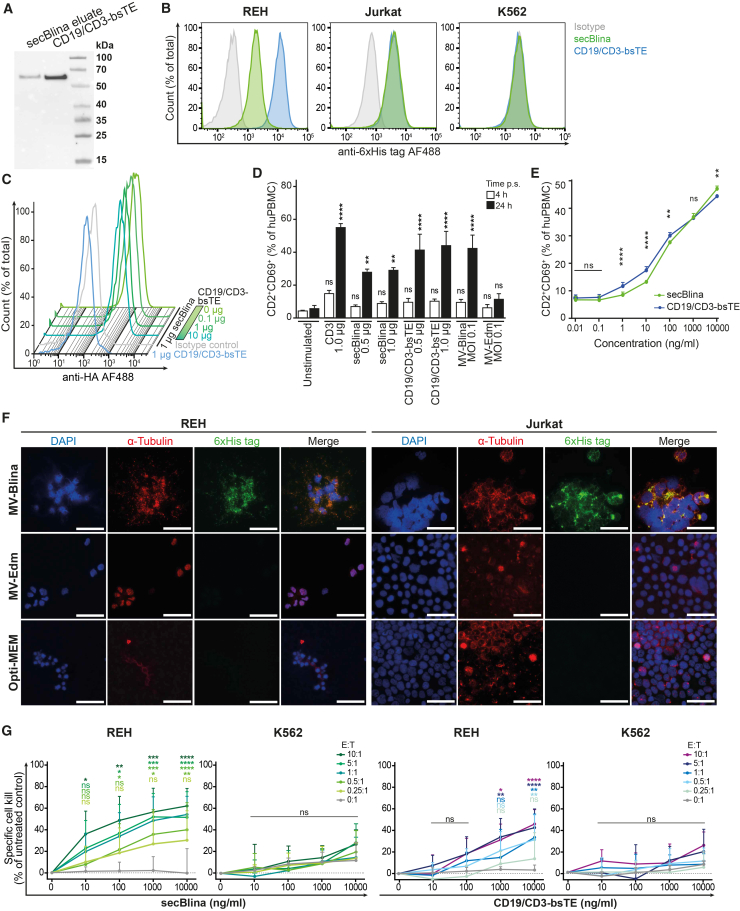
MV-Blina infected cells secrete target-binding secBlina, which initiates ALL cell kill more efficiently than CD19/CD3-bsTE (A) Vero cells secrete secBlina. secBlina was harvested from the supernatant of infected Vero cells, followed by purification and concentration. Eluate (2 μg protein) was detected via His-tag by Western Blot in comparison to the commercial CD19/CD3-bsTE (1.8 μg). (B) Purified and concentrated secBlina binds specifically to target cells. Binding to target cells was confirmed by flow cytometry using CD3^−^CD19^+^ REH, CD3^+^CD19^-^ Jurkat, and CD3^−^CD19^-^ K562 cell lines incubated with secBlina. Binding of secBlina to target cells was detected by flow cytometry via a His-tag antibody. (C) secBlina strongly binds to REH target cells. The REH cell line was first incubated with 1 μg secBlina. Increasing concentrations of CD19/CD3-bsTE were then added to displace cell-bound secBlina. Cell-bound secBlina was detected via an HA tag antibody by flow cytometry. (D) T cells in human PBMCs are activated by secBlina. Pooled PBMCs were stimulated with an (E) T cells are activated by secBlina during co-culture of PBMCs with REH cells. Pooled PBMCs were co-cultured with REH cells in a 1:1-ratio for 24 h while being treated with secBlina or the CD19/CD3-bsTE. Activated CD2^+^CD69^+^ T cells were detected by flow cytometry. (F) MV-Blina infected leukemia cells produce and secrete secBlina. REH and Jurkat cells are shown 72 h post MV infection or Opti-MEM control. secBlina was detected by His-tag IF staining. Scale bars 50 μm. (G) secBlina effectively induces REH target cell kill compared to CD19/CD3-bsTE, whereas K562 control cells are unaffected. Target (T) cells REH (left panels) and negative control cells K562 (right panels) were incubated with secBlina (green) or CD19/CD3-bsTE (blue) for 24 h at indicated concentrations in the presence of PBMCs (effector; E) at different E:T ratios. Specific cell kill was determined by flow cytometry. Results in A, B, C, and F are shown as representatives of three independent experiments. Data are shown as mean ± SD of *n* = 3 (D, E) or *n* = 5 (G) independent experiments. Statistical analysis was performed using two-way ANOVA with Dunnett’s correction. ns, not significant; ∗, *p* < 0.05; ∗∗, *p* < 0.01; ∗∗∗, *p* < 0.001; and ∗∗∗∗, *p* < 0.0001.

To assess the secretion and specific binding of secBlina, secBlina was concentrated and purified from the supernatant of infected Vero producer cells (Figure 2A), qRT-PCR demonstrated the absence of MV genes, confirming that the preparation was devoid of MV (Figure S1). Purified secBlina bound to different target cell lines, as detected via its His-tag, comparable to commercial CD19/CD3-bsTE (Figure 2B). In contrast, the CML cell line K562 (CD3^-^ and CD19^-^) showed no binding, underscoring the specificity of secBlina. Displacement studies were conducted to assess the strength of secBlina binding to REH B-cell precursor ALL cells (Figure 2C). To this end, REH cells were first incubated with 1 μg secBlina, followed by increasing concentrations of a research-grade analog of the CD19/CD3-bsTE blinatumomab to displace cell-bound secBlina. No displacement was evident, showing strong binding of secBlina to the leukemic blasts. Given that the binding of bsTE to CD3 activates T cells, we evaluated the activation potential of secBlina using pooled PBMCs from healthy donors. secBlina demonstrated activation levels comparable to CD19/CD3-bsTE or purified CD3 antigen (Figure 2D). A co-culture assay using REH and pooled PBMCs, a constant 1:1 effector-to-target ratio, and a wide titration range of both secBlina and the CD19/CD3-bsTE revealed comparable levels of T cell activation across all concentrations tested, thereby confirming equivalent functional activity of the two bispecific antibodies (Figure 2E). REH cells markedly expressed secBlina 96 h post-infection with MV-Blina (Figure 2F). Expression of secBlina protein in infected cells became detectable already at 24 h post-infection, suggesting that the secretion of secBlina begins no later than this time point (Figure S2).To investigate specific cytotoxicity, REH and K562 cells were co-cultured with pooled PBMCs at different ratios and treated with increasing concentrations of secBlina or CD19/CD3-bsTE (Figure 2G). Remarkably, secBlina exhibited superior efficacy in killing leukemic REH cells compared to CD19/CD3-bsTE, while not being cytotoxic against negative control K562 cells, indicating the specificity of secBlina. Collectively, these findings demonstrate that secBlina retains functional activity and efficacy comparable to commercial counterparts. Thus, MV-Blina infected ALL target cells secrete biologically active secBlina.

### Measles virus-Blina is variably cytotoxic in acute lymphoblastic leukemia cell lines and patient-derived xenografts, and its effect is enhanced by secBlina

**Figure 3:**
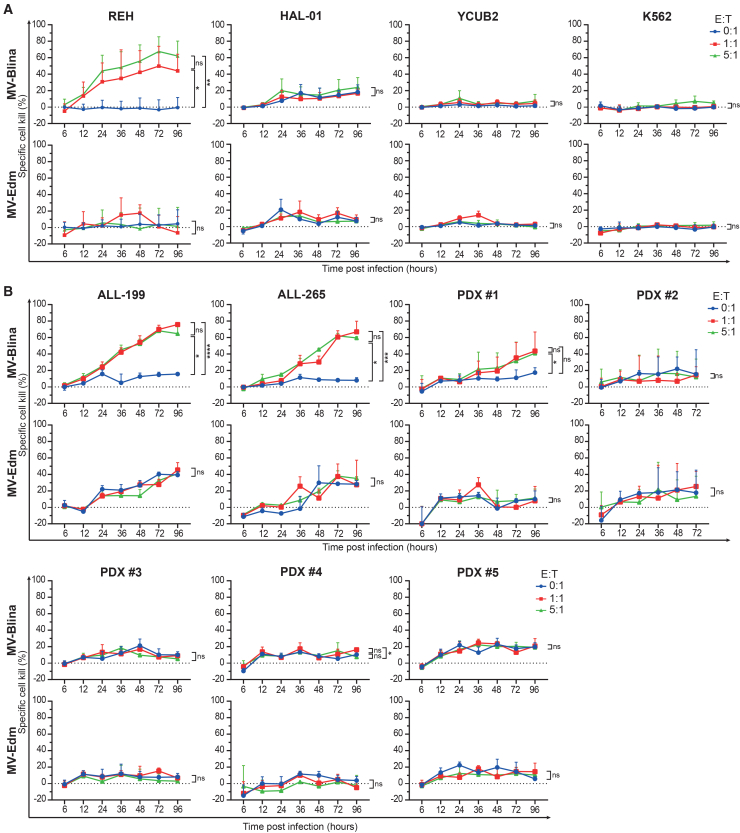
secBlina-mediated ALL cell kill in the presence of PBMCs (A) Effective but heterogeneous killing of target cell lines upon MV-Blina treatment. Target cell lines (T) were infected with MV at MOI 0.1 and co-cultured with pooled PBMCs (effectors, E) of healthy donors at different E:T ratios for the indicated time points. Specific cell kill was measured by flow cytometry. Results are shown as mean ± SD of *n* = 5 independent experiments. (B) PDX can be killed by secBlina-enhanced cytotoxic effect independent of their risk status. Target PDX cells were infected with MV and co-cultured with pooled PBMCs (effectors, E) of healthy donors at different E:T ratios for the indicated time points. Specific cell kill was measured by flow cytometry. Results are shown as mean ± SD of *n* = 3 or *n* = 4 independent experiments. Statistical analysis was performed using two-way ANOVA with Tukey’s correction. ns, not significant; ∗, *p* < 0.05; ∗∗, *p* < 0.01; ∗∗∗, *p* < 0.001; and ∗∗∗∗, *p* < 0.0001. T, target cells; E, effector cells; PDX, patient-derived xenografts.

To demonstrate secBlina-mediated leukemic cell kill, a panel of ALL cell lines and patient-derived xenografts (PDXs; Table S1) were infected with MV-Blina at MOIs of 0.1 and 1.0 and co-cultured with pooled healthy PBMCs at varying effector-to-target (E:T) cell ratios. Independent of their underlying genetic alterations, ALL cell lines exhibited heterogeneous responses in target cell killing following MV-Blina treatment (Figure 3A). REH cells demonstrated pronounced E:T ratio- and time-dependent cell killing mediated by secBlina. Among TCF3-HLF-positive cell lines, HAL-01 showed a trend toward enhanced cell killing compared to MV-Edm, whereas YCUB2 was resistant. In the PDX models, significant albeit hetrogeneous, E:T ratio- and time-dependent cytotoxic effects were observed following MV-Blina infection in the medium-risk models ALL-199 and PDX #1, as well as in the high-risk model ALL-265 (Figure 3B; Table S1). These effects were even more pronounced at MOI 1.0 (Figure S3). Of note, secBlina enhanced MV-induced cell kill. In contrast, no specific cytotoxicity was detected in high-risk PDX #2 and three other PDXs, regardless of MOI. Additional experiments were performed with three PDX on OP9 murine feeder cells to decrease unspecific apoptosis of PDX induced by the stress of *ex vivo* culture conditions. In all these PDXs, an additive response to MV-Blina was detected, including in PDX #2, which initially showed no response (Figure S4). This suggests that the more realistic conditions of PDX culture on feeder cells lead to a more pronounced response to MV-Blina. Thus, secBlina provides a distinct and additive cytotoxic effect beyond MV-Blina alone, enhancing overall anti-leukemic activity against some but not all ALL cell lines and PDXs independent of their risk status.

### Enhanced survival of acute lymphoblastic leukemia-bearing mice with measles virus-Blina compared to non-recombinant measles virus

**Figure 4:**
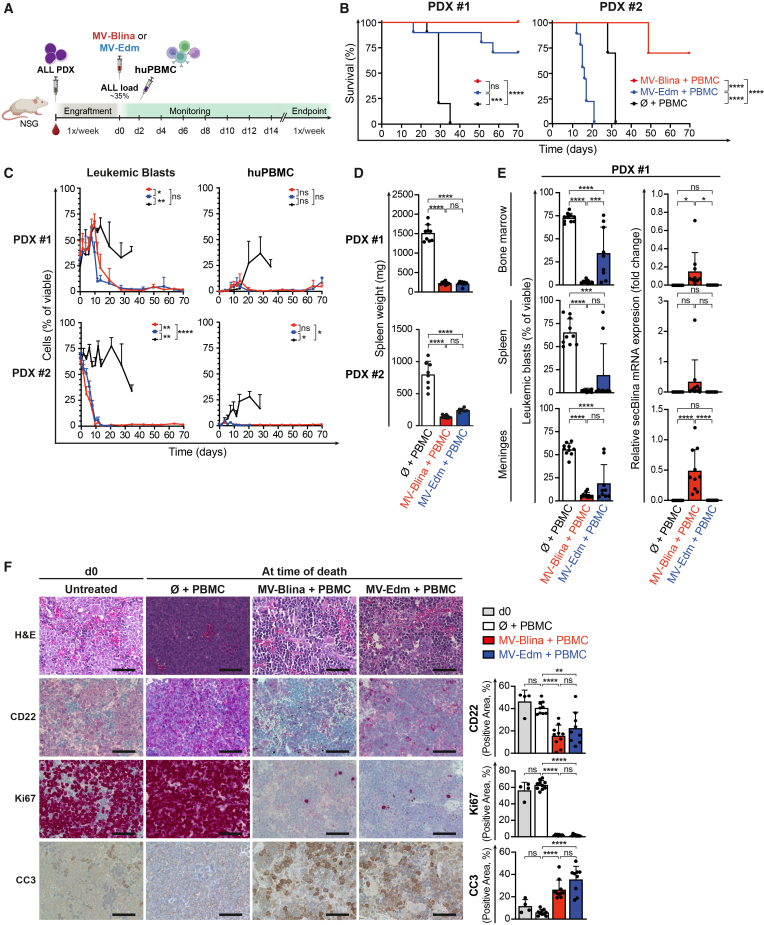
MV-Blina therapy significantly prolongs the survival of ALL bearing mice (A) Treatment scheme. Two PDX models were established in NSG mice by i.v. injection to induce ALL. At a leukemic load of approximately 35%, mice received treatment i.v. with MV (2.5 × 10^5^ TCID_50_/g) or control. Two days after infection, mice were injected with healthy human PBMCs. (B) Superior survival of PDX #2 with MV-Blina compared to MV-Edm. PDX #1 and #2 were treated either with MV-Blina and PBMC (*n* = 10) or MV-Edm and PBMC (*n* = 10) or control with PBMC only (*n* = 10) and observed for a maximum of 70 days. (C) MV-Blina treatment decreases leukemic load. Leukemic blasts (msCD45^−^huCD19^+^huCD45^dim^) and human PBMC (msCD45^−^huCD19^+^huCD45^bright^) in peripheral blood were monitored by flow cytometry at the indicated time points. (D) Marked reduction of spleen weight in PDX upon MV-Blina treatment. At the time of death, a complete necropsy was performed, and spleen weight was determined. (E) Significant reduction of leukemic blasts by MV-Blina in ALL compartments. At the time of death, leukemic blasts (msCD45^−^huCD19^+^huCD45^dim^) were detected by flow cytometry in bone marrow, spleen, and meninges. Replication of secBlina was detected by qRT-PCR and calculated by the 2^−ΔΔCt^ method, displaying the fold change relative to huActin and huGAPDH. (F) MV-Blina reduces leukemic blasts in the spleen by decreasing proliferation and increasing apoptosis. Spleens of untreated mice at therapy start (d0, *n* = 4) and treated mice (MV-Blina and PBMC, *n* = 10; MV-Edm and PBMC, *n* = 9; PBMC-only control, *n* = 9) were formalin-fixed, paraffin-embedded, and subsequently stained for H&E, CD22, Ki67, or cleaved Caspase-3 (CC3). Positive area was determined using ImageJ. Scale bars 50 μm. Statistical analysis was performed using Mantel-Cox log rank test (B), one-way ANOVA with Tukey’s multiple comparisons test (C-F). ns, not significant; ∗, *p* < 0.05; ∗∗, *p* < 0.01; ∗∗∗, *p* < 0.001; and ∗∗∗∗, *p* < 0.0001.

To evaluate the therapeutic potential of MV-Blina *in vivo*, two PDX models were selected based on their *in vitro* response: PDX #1, which showed robust specific cell killing, and PDX #2, which was largely MV-resistant (Figure 3B). The *in vivo* experiment is outlined in Figure 4A. Leukemic blasts and human PBMCs in mouse peripheral blood were distinguished by cell surface marker expression levels (Figure S5). MV-Blina significantly prolonged survival compared to MV-Edm in mice engrafted with PDX #2 and showed a trend toward greater efficacy in mice with PDX #1, both with and without the co-administration of PBMCs (Figures 4B and S6B). Surprisingly, MV-Edm reduced the survival of PDX #2-bearing mice compared to the control group, for reasons that remain unknown. Increasing the number of mice for Figures 4B and 7 might have clarified borderline findings within these experiments.

Of note, histopathological analysis excluded graft-versus-host disease, which may have impacted survival (Figure S7). MV-Blina decreased leukemic burden (Figure 4C) and significantly improved clinical scoring parameters, including general health status and signs of disease-related stress (Figure S6A). However, MV treatment also reduced PBMC levels in peripheral blood, albeit PBMCs remained detectable at the time of death in PDX #1 (Figure 4C). MV-Blina reduced the weight of the spleen (Figure 4D) and of the liver, lung, and kidneys (Figure S6C) in both PDX models, suggesting clearance of leukemic blasts. Indeed, MV-Blina reduced leukemic load across all sites of leukemic infiltration, including the CNS, a site typically protected by the blood-brain barrier, with increased efficacy compared to MV-Edm. This was supported by both blast count and mRNA expression analyses (Figures 4E and S6D). MV-Blina reduced splenic CD22^+^ leukemic blasts by decreasing Ki67-defined proliferation and increasing apoptosis detected by cleaved caspase-3 (Figure 4F).

Collectively, these results demonstrate that MV-Blina can control ALL in mice, including CNS involvement, with significantly greater efficacy than MV-Edm in the PDX #2 model and a trend toward improved efficacy in the PDX #1 model.

### Measles virus-Blina is not toxic to neuronal cells *in vitro*

**Figure 5:**
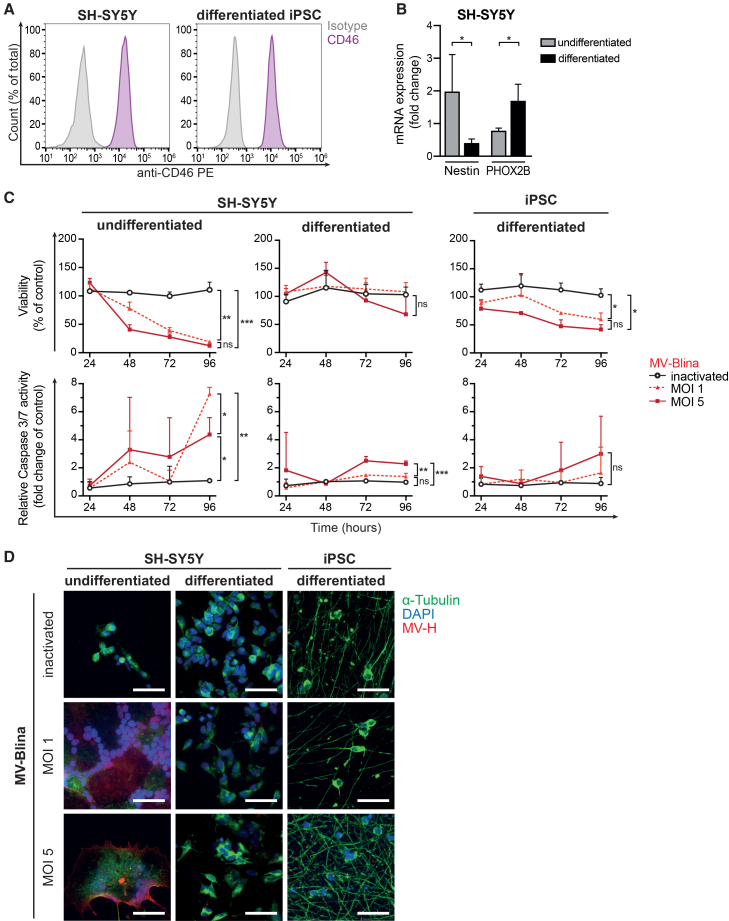
No toxicity of MV-Blina in neuronal cells *in vitro* (A) SH-SY5Y tumor cells and human iPSC-derived neurons express the same level of measles virus entry receptor CD46. The neuroblastoma cell line SH-SY5Y and differentiated iPSC (human iPSC derived glutamatergic neurons) express MV entry receptor CD46, as determined by flow cytometry. Results are shown as representatives of 3 independent experiments. (B) Neuronal expression pattern of differentiated SH-SY5Y cells. SH-SY5Y cells were seeded on poly-D-lysine and either differentiated in medium containing 5 μM ATRA and 5% FBS or cultured under normal conditions for 11 days. Expression of mRNA of the neuronal stem cell marker Nestin and the early neuronal differentiation marker PHOX2B was determined by qRT-PCR, and fold change was calculated by the 2^−ΔΔCt^ method relative to huActin. Results are shown as means ± SD of *n* = 4 independent experiments. (C) Compared to undifferentiated cells, fewer neuronally differentiated cells undergo apoptosis at a lower MOI of 1. Undifferentiated and differentiated SH-SY5Y and differentiated iPSC neurons were inoculated with either Opti-MEM (untreated control) or inactivated MV-Blina or MOI 1 or MOI 5 for different time points as indicated. Viability and apoptosis relative to untreated control were measured by CellTiter-Glo assay and Caspase-Glo 3/7 assay, respectively. Results are shown as means ± SD of *n* = 4 (SH-SY5Y) or *n* = 3 (iPSC) independent experiments. (D) No cell death and reduced syncytia formation of differentiated neuronal cells upon MV-Blina infection. Cells were stained after 96 h for α-Tubulin (green), DAPI (blue), and MV-H (red). Results are shown as representatives of *n* = 4 (SH-SY5Y) or *n* = 3 (iPSC) independent experiments. Scale bars 50 μm. Statistical analysis was performed using unpaired *t* test (B) and two-way ANOVA with Tukey’s multiple comparisons test (C). ns, not significant; ∗, *p* < 0.05; ∗∗, *p* < 0.01.

**Figure 6:**
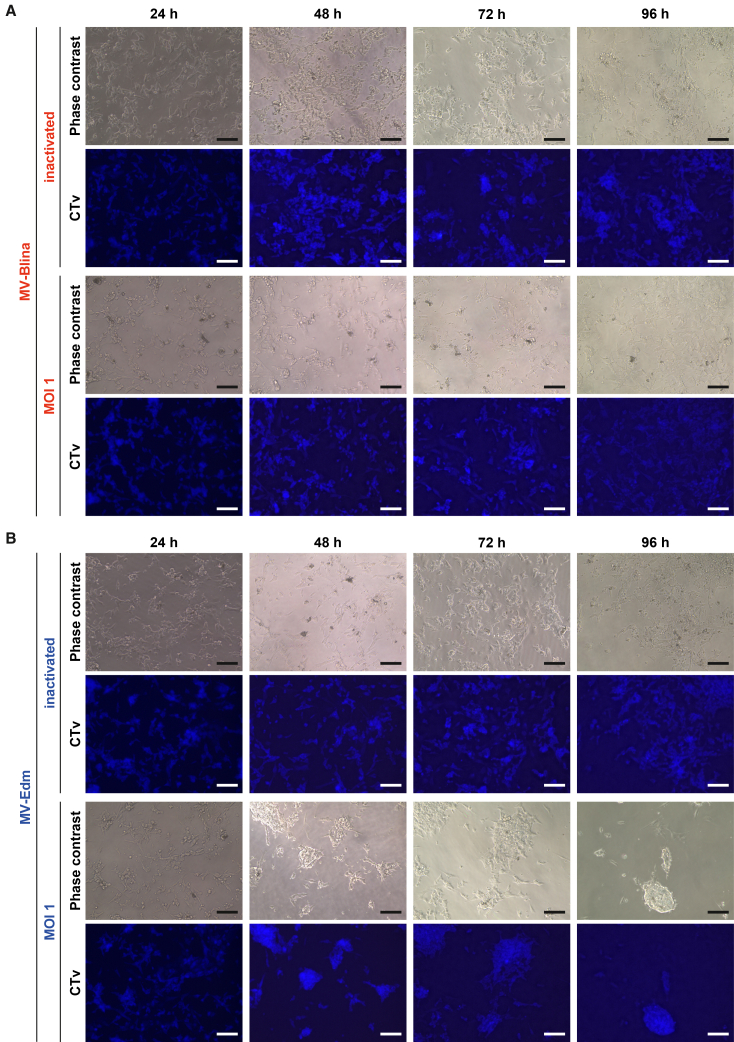
No neuronal toxicity of MV-Blina in differentiated SH-SY5Y cells in the presence of immunologically active and secBlina secreting cells (A) Neuronal-like SH-SY5Y cells exhibit no cytotoxic effect. CTv pre-stained SH-SY5Y cells were seeded on poly-D-lysine and differentiated in DMEM-based medium containing 5 μM ATRA and 5% FBS for 11 days. On day 12, SH-SY5Y were co-cultured with 24 h post-infected REH target (T) cells and pooled PBMC of healthy donors (E, effector) in an E:T ratio of 1:1 and inoculated with inactivated MV-Blina or at an MOI of 1.0. Differentiated SH-SY5Y were further co-cultured for the indicated time points. Cytotoxic and cytopathic effects were observed by microscopy. (B) Parental MV-Edm induces syncytia formation and cell death. SH-SY5Y cells were treated as described in A. Inoculation was performed using parental MV-Edm, inactivated or at an MOI of 1.0, respectively. Representatives of *n* = 5 are shown. Scale bars represents 100 μm. CTv, CellTrace Violet; E, effector cells; and T, target cells.

Given the potential for MV-induced neurotoxicity,^43^ *in vitro* assays for neurotoxicity of MV-Blina were performed. The human SH-SY5Y neuroblastoma cell line was used as a model both in its native undifferentiated state and after differentiations with all-trans retinoic acid (ATRA). Additionally, human iPSC-derived glutamatergic neurons were included as a non-tumor neuronal model. To confirm susceptibility to MV infection, the expression of the viral entry receptor CD46 was verified in these cells (Figure 5A). Differentiated SH-SY5Y cells exhibited expression profiles consistent with neuronal lineage (Figure 5B). MV-Blina significantly reduced the viability of undifferentiated SH-SY5Y cells (Figure 5C). Correspondingly, caspase-3/7 activity was significantly increased in these cells. In contrast, ATRA-differentiated SH-SY5Y and iPSC-derived neuronal cells showed minimal or no reduction of viability and no caspase activation at an MOI of 1. By comparison, MV-Edm treatment caused toxicity in both tumor and neuronal models (Figure S8A). Immunofluorescence analysis confirmed these findings, revealing no apparent toxicity and reduced syncytia formation in differentiated neuronal cells upon MV-Blina treatment (Figure 5D), whereas MV-Edm treated cells showed pronounced cytopathic effects (Figure S8B). Moreover, co-culture of differentiated SH-SY5Y cells with MV-Blina-inoculated REH target cells in the presence of PBMCs revealed no detectable toxicity (Figure 6A). Both inactivated and virulent MV-Blina were non-toxic and did not induce syncytia formation or reduce cell numbers, regardless of whether PBMCs were included in the co-culture (Figure S9). In contrast, parental MV-Edm elicited a conspicuous cytopathic effect (Figure 6B). Minor morphological changes observed in SH-SY5Y cells at 96 h post-co-culture with MV-Blina were attributable to the use of RPMI 1640 medium, which was optimized for REH cells and PBMCs but is suboptimal for neuronal cells. Taken together, these results demonstrate that MV-Blina is not toxic to differentiated neuronal cells.

### No toxicity of systemic Measles virus-Blina

**Figure 7:**
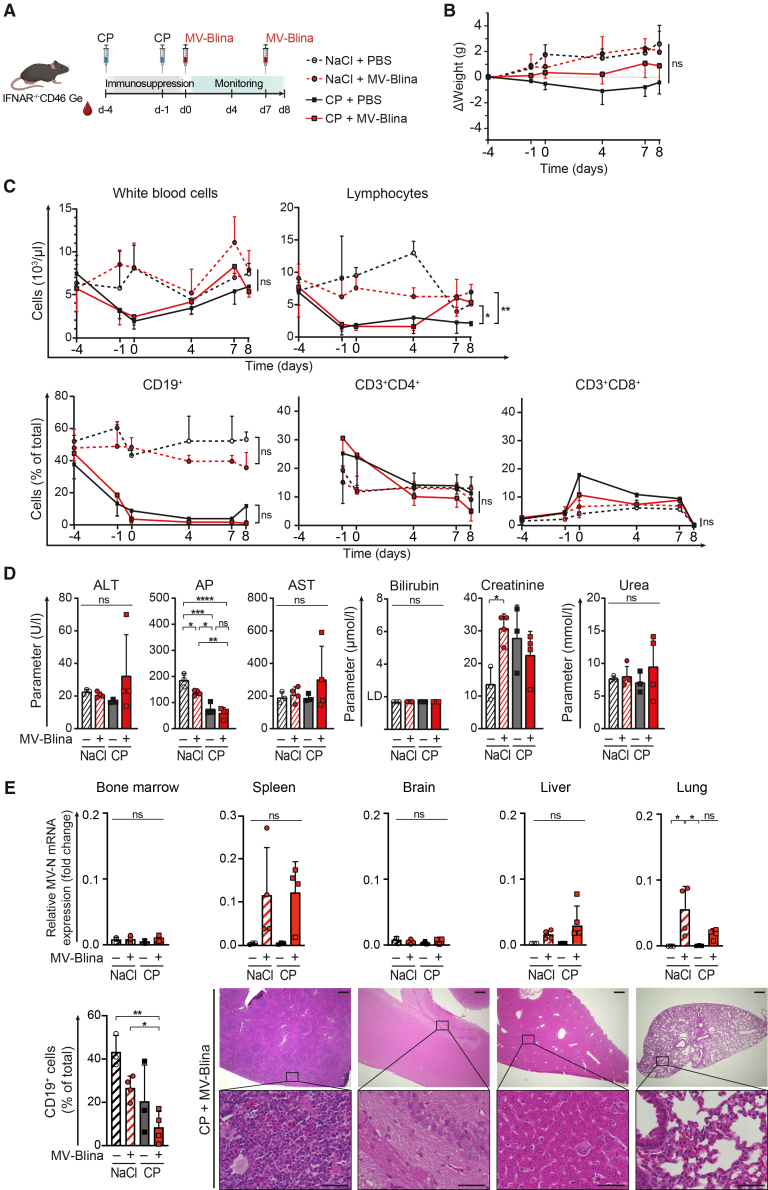
No acute toxicity of oncolytic MV-Blina *in vivo* (A) Treatment scheme. Young (28–42 days old) IFNAR^−/−^ CD46 Ge mice were injected i.v. with cyclophosphamide (CP, 150 mg/kg, solid lines) or control (0.9% NaCl, dashed lines) at day −4 and day −1. MV-Blina (1.4 × 10^10^ TCID_50_/kg, red) or control (PBS, black) were administered twice on day 0 and day 7. Mice were sacrificed on day 8 (CP, *n* = 3; CP with MV, *n* = 4; NaCl, *n* = 3; NaCl with MV-Blina, *n* = 4). (B) No weight loss. (C) Significant B-cell depletion after two i.v. injections of CP. White blood cells and lymphocytes of peripheral blood were assessed. Percentages of msCD19^+^ B-cells, msCD3^+^CD4^+^, and msCD3^+^CD8^+^ T cells in peripheral blood were determined by flow cytometry. (D) No hepatic or renal short-term toxicity. Serum hepatic parameters (ALT, alanine aminotransferase; AST, aspartate aminotransferase; AP, alkaline phosphatase; bilirubin) and renal parameters (creatinine, urea) were evaluated on day 8. (E) MV-Blina infects the liver, lung, and spleen but not the bone marrow and brain. RNA was isolated from the indicated tissues at the time of sacrifice. Expression of measles virus mRNA (MV-N) was detected using qRT-PCR, and fold change was calculated by the 2^−ΔΔCt^ method relative to msActin and msGusb. The percentage of msCD19^+^ cells in bone marrow was assessed by flow cytometry. Representative tissue sections stained for H&E are shown for the group subjected to CP and treated with MV-Blina. Scale bars represent 300 μm (upper panels) and 50 μm (lower panels). All results are shown as means ± SD. Statistical analysis was performed using two-way ANOVA with Tukey’s multiple comparisons test (B-C) and one-way ANOVA with Tukey’s comparisons test (D-F). ns, not significant; ∗, *p* < 0.05; ∗∗, *p* < 0.01; ∗∗∗, *p* < 0.001; and ∗∗∗∗, *p* < 0.0001.

Having demonstrated the absence of cytotoxic effects on neuronal cells *in vitro*, systemic short- and long-term toxicity of MV-Blina was assessed *in vivo*. To determine the acute toxicity of MV-Blina, immunocompromised mice with IFNAR knockout and transgenic for CD46 were used (Figure S10A).^44^ Young (28–42 days old) mice were treated with immunosuppressive CP to mimic the young age and potential immunocompromise encountered in pediatric patients with ALL. Subsequently, mice were injected twice with MV-Blina (Figure 7A). No weight loss was observed in young or older (43–56 days old) mice (Figures 7B and S11A, respectively). No other clinical signs of toxicity, in particular no neurological deficits, were apparent. CP administration resulted in the expected moderate lymphocytopenia and a reduction of CD19+ B-cells (Figures 7C and S11B) and shifted hematopoiesis toward the extramedullary compartment (Figures S11F and S10). No hepatic or renal toxicity was found by clinical chemistry (Figure 7D). Importantly, no histological signs of toxicity were evident in the brain, liver, and lung (Figures 7E and S12). Similar results were obtained in young adult immunocompromised mice (Figure S11). MV-Blina infection was detected in the liver, lung, and spleen, but not in bone marrow and, importantly, in the brain, as determined by MV-N mRNA expression (Figure 7E). Taken together, MV-Blina does not exert short-term toxicity.

**Figure 8:**
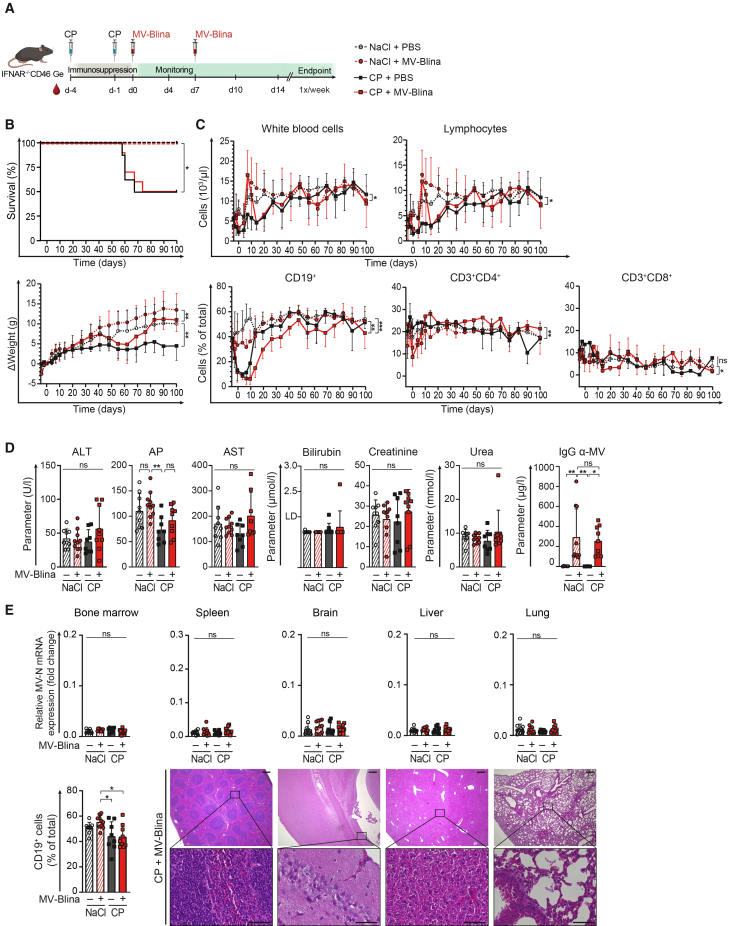
No long-term toxicity of MV-Blina *in vivo* (A) Treatment scheme. Young (28–42 days old) IFNAR^−/−^CD46tg mice were injected i.v. with cyclophosphamide (CP, 150 mg/kg, solid lines) or control (0.9% NaCl, dashed lines) at day −4 and day −1. MV-Blina (1.4 × 10^10^ TCID_50_/kg, red) or control (PBS, black) was administered twice on day 0 and day 7. Mice were observed for a maximum of 100 days (CP, *n* = 8; CP with MV, *n* = 10; NaCl, *n* = 9; NaCl with MV-Blina, *n* = 10). (B) No death and no weight loss. Survival and body weight changes as long-term toxicity indicators were monitored. (C) Blood cell counts after MV-Blina and recovery from CP-induced B-cell depletion. Peripheral white blood cells and lymphocytes, as well as msCD19^+^ B-cells, msCD3^+^CD4^+^ T cells, and msCD3^+^CD8^+^ T cells were assessed as in Figure 6. (D) Absence of hepatic or renal long-term toxicity with elevated MV-specific antibody levels. Serum hepatic parameters (ALT, AP, AST, bilirubin) and renal parameters (creatinine, urea) were assessed at the time of death. MV specific IgG antibodies were measured by ELISA. (E) No detection of MV-N and no histopathological signs of toxicity in various organs. MV-N mRNA expression was determined by qRT-PCR as described in Figure 6. The percentage of msCD19^+^ cells in bone marrow was assessed by flow cytometry. Representative tissue sections stained for H&E are shown for the group subjected to CP and treated with MV-Blina. Scale bars 300 μm (upper panels) and 50 μm (lower panels). All results are shown as means ± SD. Statistical analysis was performed using log rank Mantel-Cox test (B), two-way ANOVA with Tukey’s multiple comparisons test (B-C), and one-way ANOVA with Tukey’s comparisons test (D-F). ns, not significant; ∗, *p* < 0.05; ∗∗, *p* < 0.01; ∗∗∗, *p* < 0.001; and ∗∗∗∗, *p* < 0.0001.

Long-term toxicity was assessed by evaluating survival in young mice receiving MV-Blina, with or without prior cyclophosphamide (CP) pretreatment (Figure 8A). Despite severe lymphopenia, no signs of long-term toxicity, particularly no neurological deficiencies, were observed. Survival was unaffected, and body weight did not decrease (Figure 8B). As expected, CP administration led to a depletion of CD19^+^ B-cells (Figure 8C), which gradually recovered over time. Interestingly, MV-Blina infection induced a transient increase in white blood cell counts shortly after administration. No significant MV-Blina-mediated lymphopenia was observed (Figure 8C). Importantly, there was no evidence of hepatic or renal toxicity. MV-specific antibodies were induced (Figure 8D), in line with a vaccination effect. Additionally, extramedullary hematopoiesis resolved, as indicated by normal spleen and liver weights (Figure S13A). No MV-N transcripts were detected in organs, in particular not in the brain (Figure 8E), and histopathological analyses of multiple organs, most importantly of the brain, revealed no signs of tissue damage (Figure S13B).

Taken together, MV-Blina in a clinically relevant dose does not exert short- or long-term toxicity even in young and immunocompromised mice.

## Discussion

OMVs have demonstrated efficacy in tumor models and have progressed into Phase I/II clinical trials.^21^^,^^22^^,^^23^^,^^24^^,^^25^^,^^26^^,^^27^^,^^28^^,^^29^^,^^30^^,^^31^^,^^33^^,^^34^^,^^35^^,^^36^^,^^37^^,^^45^ Intratumorally injected OMVs encoding bsTEs targeting CD3 and CEA or CD20 were effective in immunocompetent mice bearing patient-derived colorectal cancer xenografts and receiving PBMCs.^49^ In the present study, a recombinant OMV encoding a secreted CD19/CD3-bsTE (secBlina) was established. *In vitro*, secBlina bound and activated CD3^+^ T cells comparably to the commercial counterpart. Surprisingly, it exhibited stronger binding to CD19^+^ target cells than the commercial CD19/CD3-bsTE, despite the latter being reported as Blinatumomab-like by the manufacturer. secBlina not only enhanced the efficacy of the OMV against BCP-ALL *in vitro*, but also *in vivo* without inducing significant toxicity. By combining MV-induced direct leukemolysis with secBlina-mediated T cell engagement and activation, MV-Blina significantly prolonged survival in ALL-bearing mice and reduced leukemic burden, including in the CNS.

Several aspects of MV-Blina therapy against pediatric BCP-ALL warrant particular consideration. First, MV-Blina exhibited greater direct efficacy than its parental strain, MV-Edm, across multiple *in vitro* assays, notably achieving a higher replication rate despite its larger genome, even though both share an identical cloning backbone.

Second, ALL-PDX models were heterogeneous in their response to MV-Blina, both *in vitro* and *in vivo*. Several mechanisms relevant to experimental settings may explain this heterogeneity. These include the presence of partially intact type I interferon signaling, known to drive resistance to oncolytic viruses in general^52^^,^^53^ and to OMV in particular,^54^ as well as heterogeneity in MV receptor expression.^18^^,^^55^ Further mechanisms may be heterogeneous expression of inhibitory ligands such as PD-L1^56^^,^^57^ and microenvironmental factors that physically limit viral spread.^52^^,^^53^ These potential mechanisms constitute important venues for future investigations, in addition to genetic and molecular biomarkers for predicting sensitivity to MV-Blina.

Third, although secBlina demonstrated an additive effect beyond the direct cytotoxic activity of MV-Blina, this effect was not always statistically significant. This may be attributable to limited numbers and suboptimal functionality of the infused T cells, as well as to the timing of their administration. Hence, ensuring a sufficient number of functional T cells in patients will be important for clinical application.

The presence or absence of neutralizing antibodies against the measles virus will influence the clinical efficacy of MV-Blina. Between 20% and 60% of children with ALL lack protective anti-measles antibody titers,^58^^,^^59^ and may thus be susceptible to MV-Blina. In seropositive patients, strategies such as antibody depletion or delivery via cellular carriers may be necessary to bypass neutralization, enhance tumor targeting, and minimize off-target effects, as we and others have demonstrated.^60^^,^^61^^,^^62^^,^^63^^,^^64^ Notably, even in the presence of an emerging antiviral immune response, MV-Blina may retain replicative capacity and therapeutic efficacy through cell-to-cell spread, similar to what we have previously shown for MV-Edm.^34^

Repeated intravenous administration of MV-Blina may further increase therapeutic efficacy, as has been proposed for other OMVs.^47^^,^^65^ However, a single dose of 2.5 × 10^5^ TCID_50_/g body weight of MV-Blina was sufficient to eradicate ALL in our study. This dose has also been effective in patients with solid tumors, with few reported side effects.^31^

Importantly, despite using a measles-susceptible mouse model deficient in type I interferon signaling, applying additional cyclophosphamide-induced immunosuppression and administering two high-dose intravenous injections of MV-Blina, no toxicity was observed. This aligns with clinical observations of low toxicity and minimal histopathological abnormalities upon the administration of other recombinant OMVs, such as MV-s-NAP, which encodes the secretory form of *Helicobacter pylori* neutrophil-activating protein (s-NAP) and was administered intravenously as well as subcutaneously in susceptible mice.^45^ In patients with multiple myeloma, intravenous administration of a recombinant MV encoding the human iodide symporter (MV-NIS) at the maximum feasible dose of 10^11^ TCID_50_ was well tolerated.^31^ Similarly, no toxicity was observed in patients with ovarian cancer receiving intraperitoneal MV-NIS^32^ or MV-CEA.^21^

Neurotoxicity remains a particular concern for OMVs. However, minimal toxicity was observed in both neuronally differentiated SH-SY5Y human neuroblastoma cells and iPSC-derived glutamatergic neurons, even in the presence of PBMCs, whereas parental MV-Edm exhibited more pronounced toxicity. *In vivo*, MV-Edm induced neurotoxicity in immunodeficient IFNAR^−/−^ CD46 Ge mice only after intracerebral, but not intravenous, administration.^44^ Importantly, in a clinical setting, patients with glioblastoma multiforme, who received MV-CEA injections into the tumor resection cavity, did not experience neurotoxicity.^23^ Subacute sclerosing panencephalitis (SSPE) is a rare but serious delayed neurotoxic sequela of wild-type measles infection in humans.^66^ However, long-term follow-up of MV-Blina-treated mice did not reveal evidence of SSPE. Although the most sensitive model for SSPE involves the intracranial injection of MV into neonatal mice, the clinically relevant model used here, intravenous injection of MV-Blina into young immunocompromised mice, is sufficiently sensitive to suggest that the risk of SSPE is low. Nevertheless, the possibility of a higher risk cannot be excluded if OMVs were to persist within leukemic blasts in the CNS.

As a general consideration, a disease model mimicking minimal residual disease and late relapse may more closely resemble the clinical scenario in pediatric ALL and could be used to further assess the therapeutic utility of MV-Blina, compared to the florid leukemia model employed in this study. In addition, a murine-compatible MV-Blina analog would allow comprehensive toxicity studies in IFNAR^−/−^ CD46 Ge mice.

In conclusion, the results presented here support further investigation into the feasibility of MV-Blina for the treatment of relapsed or refractory B-cell precursor ALL.

## Materials and methods

### Cells and cell culture

The producer cell line Vero, the leukemic cell lines REH, Jurkat, K562, and HAL-01, as well as the neuroblastoma cell line SH-SY5Y, were obtained from DSMZ (Braunschweig, Germany). The murine feeder cell line OP9 was procured from the American Type Culture Collection (ATCC, Manassas, VA). The B-cell acute lymphoblastic leukemia (ALL) cell line YCUB2 was generously provided by Hiroaki Goto (Kanagawa Children’s Medical Center, Yokohama, Japan). Human iPSC-derived glutamatergic neurons (ioGlutamatergic Neurons, ab259259) were purchased from Abcam (Cambridge, UK). Human peripheral blood mononuclear cells (PBMCs) were isolated from the whole blood of healthy donors younger than 25 years by Ficoll density gradient centrifugation using BioColl (Bio&Sell, Feucht, Germany). Vero and SH-SY5Y cells were maintained in DMEM with 10% fetal bovine serum (FBS), 2 mM L-glutamine and 100 U/mL penicillin and 100 μg/mL streptomycin (all from Gibco, Thermo Fisher Scientific, Waltham, MA). Leukemic cell lines and PBMCs were cultured in RPMI 1640 (Gibco) supplemented with 10% FBS and 2 mM L-glutamine. Pediatric B cell ALL PDX #1 - #5 were established and provided by Lüder H. Meyer (Ulm University Medical Center, Ulm, Germany).^67^^,^^68^ The chemoinsensitive pediatric pre-B ALL patient-derived xenografts (PDX) ALL-199 and ALL-265 were generously provided by Irmela Jeremias (Helmholtz Center, Munich, Germany) and have been previously described.^69^^,^^70^^,^^71^ PDX samples were cultured *ex vivo* in RPMI 1640 supplemented with 20% FBS and 2 mM L-glutamine. The murine cell line was maintained in Alpha-MEM Eagle (PAN Biotech, Aidenbach, Germany) supplemented with 20% FBS, 2 mM L-glutamine, and 100 U/mL of penicillin and 100 μg/mL of streptomycin (all from Gibco). All cells were cultured at 37°C in a humidified atmosphere with 5% CO_2_. All cells were routinely tested for mycoplasma contamination using the MycoStrip Mycoplasma Detection Kit (Invivogen, San Diego, CA) according to the manufacturer’s recommendations.

### Generation of measles virus-bispecific T cell engager construct

The MV-Edmonston B vaccine strain, containing an additional transcription unit downstream of the H open reading frame, was used as the backbone for generating the new MV-Blina construct and was provided by C. E. Engeland (Leipzig University, Leipzig, Germany).^49^ The CD19/CD3-bsTE sequence, comprising variable heavy (VH) and variable light (VL) chain regions in the order VH(CD19)-VL(CD19)-VH(CD3)-VL(CD3) from 3′-leader to 5′-trailer and connected by artificial Gly_4_Ser peptide linkers, was obtained from Sequence 29 of Patent WO2004106381 (GenBank accession CQ967794.1). For secretion, the original signal peptide of murine Ig heavy chain V region was exchanged with the signal peptide of the human Ig heavy chain V-I region (TGGATTGGACCTGGCGGATTCTGTTCCTTGTAGCC GCAGCTACTGGGGCCCACT) and inserted downstream of the Kozak consensus sequence. An influenza virus hemagglutinin (HA) tag and a hexahistidine (His_6_) tag were additionally incorporated downstream of the signal peptide and the bsTE sequence, respectively. The construct was generated by gene synthesis (Eurofins, Ebersberg, Germany) and inserted into an additional transcription unit downstream of the H open reading frame within the MV genome. The final construct was validated by Sanger sequencing (Eurofins).

### Measles virus propagation, titration, and inactivation

The recombinant MV-bsTE virus was generated by transfecting Vero cells with antigenomic cDNA constructs as described previously.^72^^,^^73^ The MV-Edmonston B strain (VR-24, ATCC, Manassas, VA, USA) was obtained and amplified prior to experiments. For MV amplification, Vero cells were infected at a multiplicity of infection (MOI) of 0.03 and cultured at 32°C in a humidified atmosphere with 5% CO_2_. Syncytia formation as a cytopathic effect was monitored by light microscopy. When ≥90% of cells were in syncytia, virus-containing cells were scraped in Opti-MEM (Gibco), and viral particles were released by two consecutive freeze-thaw cycles. The virus-containing suspension was centrifuged (2500 × g, 15 min, 4°C) and the virus-containing supernatant was stored in aliquots at −80°C. Virus titer was determined by the TCID_50_ assay.^74^ MV-Blina and MV-Edm were inactivated at 37°C for at least 7 days. Complete MV inactivation was confirmed by titration.

### Measles virus replication and cytotoxicity assays

To compare the replication of MV-Blina and MV-Edm, 1 × 10^5^ Vero cells per well were seeded in 12-well plates and infected in duplicates per time point at an MOI of 0.01 or 1.0 in serum-free medium, followed by incubation at 37°C in a humidified atmosphere with 5% CO_2_. The medium was replaced 3 h post-infection with medium containing 10% FBS. Infected Vero cells were scraped at the indicated time points and stored at −80°C. Viral titers of the samples were determined as described above. To assess cytotoxicity of MV-Blina and MV-Edm, 1 × 10^4^ Vero cells per well were seeded in 96-well plates and infected in sextuplicates per time point at an MOI of 0.01, 0.1, and 1.0 in serum-free medium and incubated at 37°C in a humidified atmosphere with 5% CO_2_. The medium was replaced 3 h post-infection with medium containing 10% FBS. At indicated time points, medium was exchanged with 100 μL of MTT solution (1 mg/mL, Sigma-Aldrich, Hamburg, Germany) per well and incubated at 37°C in a humidified atmosphere with 5% CO_2_. After 3 h MTT solution was replaced with 2-propanol (VWR, Darmstadt, Germany), incubated for 30 min on a plate shaker, and the absorbance was measured at 550 nm using the Infinite 200 PRO plate reader (Tecan, Männedorf, Switzerland).

### secBlina production and purification

secBlina was harvested from the supernatant of infected Vero cells. For production, 2 × 10^6^ Vero cells were seeded in 10 cm-dishes, infected with MV-Blina at MOI 1.0, and incubated at 37°C in a humidified atmosphere with 5% CO_2_. At 24 h post infection, cells were washed twice with PBS (Gibco), and medium was replaced with VP-SFM (Gibco) containing 100 U/mL penicillin/streptomycin, followed by further incubation at 37°C in a humidified atmosphere with 5% CO_2_. Cells were monitored by light microscopy until 95–100% syncytia formation was observed. The supernatant of infected cells was centrifuged (2000 × g, 15 min, 4°C), processed through 0.2 μm syringe filters (Whatman, Maidstone, UK) and subsequently concentrated by centrifugation (4000 × g, 20 min, 4°C) using Amicon Ultra-15 PLGC Ultracel-PL Membrane 10K (Merck Millipore, Billerica, MA). The concentrated supernatant was pooled and purified using Ni-NTA spin columns for 6xHis-tagged proteins (Qiagen, Hilden, Germany) according to the manufacturer’s protocol. For buffer exchange, the purified secBlina was mixed twice with six volumes of PBS, followed by centrifugation (4000 × g, 20 min, 4°C) using Amicon Ultra-2 Ultracel-10 regenerated cellulose membrane 10K (Merck Millipore). secBlina concentration was determined by BCA assay (Thermo Fisher Scientific, Waltham, MA).

### Western Blot analysis

secBlina and commercial CD19/CD3-bsTE (BPS Bioscience, San Diego, CA) were detected using mouse anti-6xHis tag antibody (clone HIS.H8, 1:1000, ab18184, Abcam) followed by a goat anti-mouse IgG (H + L) HRP secondary antibody (#1706516, 1:15000, Bio-Rad, Feldkirchen, Germany). Images were acquired using the ChemiDoc MP Imaging System (Bio-Rad).

### secBlina binding and displacement assay

A total of 1 × 10^6^ REH, Jurkat, or K562 cells were incubated with 1 μg of secBlina or commercial CD19/CD3-bsTE (BPS Bioscience) for 1 h at 4°C in the dark. Binding to target cells was assessed by flow cytometry using anti-6xHis tag AF488 antibody (1:200, ab237336, Abcam).

For the displacement assay, 1 × 10^6^ REH cells were incubated with 1 μg secBlina for 30 min, followed by increasing concentrations of a research-grade analog of the CD19/CD3-bsTE blinatumomab (BPS Bioscience) for an additional 30 min, all at 4°C. secBlina bound to target cells was detected using rabbit anti-HA antibody (clone EPR22819-101, 1:600, ab256483, Abcam) for 30 min at 4°C, followed by a goat-anti-rabbit IgG (H&L) AF488 (1:2000, ab150077, Abcam). Flow cytometry was used for detection (Attune NxT, Thermo Fisher Scientific), and data were analyzed with FlowJo software (v10.10.0, BD Biosciences, Heidelberg, Germany).

### T cell activation assay

5 × 10^5^ human PBMCs were seeded in 1 mL per well in 24-well plates. Cells were incubated overnight at 37°C in a humidified atmosphere with 5% CO_2_ in the presence of CD3 antibody (16-0037-81, Invitrogen, Life Technologies Carlsbad, CA), secBlina, CD19/CD3-bsTE (BPS Bioscience), MV-Blina, or MV-Edm. To compare T cell activation under co-culture conditions, 1.5 × 10^4^ REH and 1.5 × 10^4^ human PBMCs pooled from four healthy donors were seeded in 100 μL per well in 96-well plates and incubated with increasing concentrations of secBlina or CD19/CD3-bsTE (BPS Bioscience) for 24 h at 37°C in a humidified atmosphere with 5% CO_2_. T cell activation was determined by flow cytometry using a mouse anti-human CD2 APC antibody (clone RPA-2.10, 1:100, 17-0029-42, ebioscience, Invitrogen) and a mouse anti-human CD69 Pacific Blue antibody (clone FN50, 1:100, 310919, biolegend, San Diego, CA). Flow cytometry was performed using the Attune NxT Flow Cytometer (Thermo Fisher Scientific) and data were analyzed with FlowJo software (v10.10.0, BD Biosciences).

### secBlina secretion detection

A total of 1 × 10^6^ REH or 5 × 10^5^ Jurkat cells were seeded in 12-well plates and inoculated with MV-Blina or MV-Edm at MOI 1.0 or Opti-MEM (Gibco) at 37°C in a humidified atmosphere with 5% CO_2_. At indicated time points, cytospins were performed at 300 × g for 5 min using Shandon Cytofunnel (Thermo Fisher Scientific). Subsequently, cells were fixed in 4% paraformaldehyde (PFA; Sigma-Aldrich) for 10 min at room temperature (RT), followed by blocking in PBS with 0.1% Tween 20 (Sigma-Aldrich), 10% BSA (Serva, Heidelberg, Germany), and 1% normal goat serum (Thermo Fisher Scientific) at 4°C overnight. Cells were stained using a rabbit anti-6xHis AF488 antibody (1:200, ab237336, Abcam), a mouse anti-alpha tubulin antibody (1:200, CP06, Calbiochem, Merck Millipore), and DAPI (1:5000, Hoechst 33342, Invitrogen).

### Co-culture

To assess the specific targeting of secBlina, REH and K562 cells were pre-stained with CellTrace Violet (CTv; Thermo Fisher Scientific) according to the manufacturer’s protocol. Subsequently, CTv stained cells (REH, 1.5 × 10^4^/well; K562, 8 × 10^3^/well) were seeded in 96-well plates and incubated with the indicated concentrations of secBlina or CD19/CD3-bsTE (BPS Bioscience) in the presence of pooled human PBMCs from four healthy donors at indicated effector:target (E:T) ratios for 24 h at 37°C in a humidified atmosphere with 5% CO_2_. Dead cells were stained with 7-AAD (BD Biosciences) as a viability control. Flow cytometry was performed using the Attune NxT Flow Cytometer (Thermo Fisher Scientific), analyzed with FlowJo software (v10.10.0, BD Biosciences) and specific cell kill was calculated relative to the untreated control as percentage (specific lysis (%) = 100% (CTv^+^ viable cells of untreated control at respective E:T ratio) – viable % (CTv^+^7AAD^−^)). To determine the secBlina-mediated cell kill upon MV infection, leukemic cell lines (1.5 × 10^4^/well), control K562 cells (8 × 10^3^/well), and PDX samples cultured *ex vivo* (1.5 × 10^4^/well) were prestained with CTv as described above and seeded in 96-well plates. Cells were inoculated for 3 h with MV-Blina or MV-Edm at MOI 0.1 or 1.0 in serum-free medium at 37°C in a humidified atmosphere with 5% CO_2_. Human PBMCs from healthy donors were pooled and added to infected target cells and their medium at indicated E:T ratios in a final volume of 100 μL medium containing 10% (v/v) FBS. For the co-culture of fragile PDX, 3 × 10^3^ OP9 cells were seeded per 96-well. PDX #2, #3, and #5 were pre-stained with CTv as described above, cultured (1.5 × 10^4^ cells/well) on pre-seeded OP9 cells and inoculated with MV-Blina or MV-Edm at an MOI of 1.0 in AIM V medium (Gibco) at 37°C in a humidified atmosphere with 5% CO_2_. Human PBMCs from healthy donors were pooled and added at indicated E:T ratios in a final volume of 100 μL AIM V medium. At the indicated time points post-infection, specific cell kill was determined by flow cytometry using the Attune NxT Flow Cytometer (Thermo Fisher Scientific), analyzed with FlowJo software (v10.10.0, BD Biosciences) and calculated as described above.

### Neurotoxicity assessment *in vitro*

Human iPSC-derived glutamatergic neurons (ioGlutamatergic Neurons, ab259259) were differentiated according to the manufacturer’s protocol. After thawing, iPSC-derived cells were seeded onto poly-D-lysine- (Merck, Darmstadt, Germany) and Geltrex- (Thermo Fisher Scientific) coated 96-well plates (5 × 10^4^ cells/cm^2^ in triplicates) or 8-well Falcon chamber slides (3.5 × 10^4^ cells/cm^2^ in duplicates; Fisher Scientific) and further differentiated according to the manual using Neurobasal medium (Gibco), 1× Glutamax (Thermo Fisher Scientific), 25 μM 2-mercaptoethanol (Gibco), 2% B27 (Gibco), 10 ng/mL NT3 (PeproTech, Cranbury, NJ), 5 ng/mL BDNF (PeproTech), 1 μg/mL doxycycline (Clonetech, Mountain View, CA) and 10 μM DAPT (Bio-Techne, Wiesbaden, Germany). The SH-SY5Y cell line was differentiated into neuronal cells using 5 μM all-trans retinoic acid (ATRA; Sigma-Aldrich) in DMEM containing 5% FBS and seeded onto poly-D-lysine- and Geltrex-coated 96-well (5 × 10^4^ cells/cm^2^ in triplicates) or 8-well chamber slides (3.5 × 10^4^ cells/cm^2^ in duplicates). After 11 days of differentiation, the mRNA expression of PHOX2B and Nestin was determined by qRT-PCR to confirm a differentiated state. Undifferentiated SH-SY5Y cells were seeded in 96-well plates (1.5 × 10^5^ cells/cm^2^ in triplicate) and in 8-well chambers (1.5 × 10^5^ cells/cm^2^ in duplicates). Neuronal cells were inoculated in appropriate medium with MV-Blina or MV-Edm at an MOI of 1.0 and 5.0 or treated with the equivalent volume of inactivated MV corresponding to the highest MOI. Cells were incubated for the indicated time points at 37°C in a humidified atmosphere with 5% CO_2_.

Cells treated in 8-well chambers were stained by immunocytochemistry, while cells in 96-well plates were analyzed for cell viability using the CellTiter-Glo assay and for apoptosis induction using the Caspase-Glo 3/7 assay (both Promega, Walldorf, Germany), according to the manufacturer’s protocol. Luminescence signals were quantified using the Infinite 200 PRO plate reader (Tecan).

To evaluate the toxicity of MV-Blina in combination with secBlina secretion by BCP-ALL target cells, SH-SY5Y cells were pre-stained with CTv, differentiated as described above, and seeded (1 × 10^6^ cells/well) in 24-well plates at 37°C in a humidified atmosphere with 5% CO_2_. REH cells (1 × 10^5^ cells/well) and pooled human PBMC of healthy donors (1 × 10^5^ cells/well) were inoculated with MV-Blina or MV-Edm at an MOI of 1.0 or treated with the equivalent volume of inactivated MV corresponding to the highest MOI. After 24 h post-infection of the PBMC:REH co-culture and a total SH-SY5Ydifferentiation period of 11 days, the differentiation DMEM-based medium was replaced by PBMC:REH co-culture using RPMI 1640-based medium. Co-cultures were incubated for the indicated time points. Microscopic images were acquired using a Keyence BZ-9000E microscope and analyzed using BZ-II Viewer and BZ-II Analyzer v2.1 software (Keyence, Neu-Isenburg, Germany).

### Mouse experiments

All mouse experiments were approved by the regional council according to the German Animal Welfare Act (Regierungspräsidium Tübingen, Reg.Nr. 1501) and were performed in line with state and institutional guidelines. Mice were housed in individually ventilated cages with a maximum of five individuals under specific pathogen-free conditions in the Animal Research Center, Ulm.

To test ALL therapy efficacy, female NSG mice (NOD.Cg-Prkdc^SCID^ Il2rg^tm1Wjl^/SzJ, Charles River, Sulzberg, Germany) were used. To evaluate toxicity *in vivo*, IFNAR^−/−^ CD46 Ge strain mice were kindly provided by Michael D. Mühlebach (Paul-Ehrlich-Institut, Langen, Germany) and Roberto Cattaneo (Mayo Clinic, Rochester, MN). This immunocompetent and MV-susceptible mouse model has been previously described.^44^ Mice were housed and bred at the Animal Research Center, Ulm. Mouse genotypes for IFNAR knockout and human CD46 were validated by PCR. CD46 expression was additionally confirmed by flow cytometry using an anti-human CD46 PE antibody (1:20, 12-0469-42, eBiosciences, San Diego, CA).

### Acute lymphoblastic leukemia therapy *in vivo*

Six-to eight-week-old NSG mice received tail vein injections of 1 × 10^7^ PDX in 100 μL D-PBS (maximum 5 mL/kg; Gibco). Mice were checked for health status and body weight and human CD19^+^CD45^+^ blasts in murine peripheral blood were assessed by flow cytometry (Attune NxT Flow Cytometer, Thermo Fisher Scientific) using rat anti-mouse CD45 PE (1:20, clone Ly5, 553081, BD Biosciences), mouse anti-human CD19 APC (1:20, clone HIB19, 563879, BD Biosciences) and mouse anti-human CD45 BV421 (1:20, clone HI30, 555415, BD Biosciences). Percentages of human blasts were determined with FlowJo software (v10.10.0, BD Biosciences). MV therapy (MV-Blina or MV-Edm) was initiated when the leukemic load (human CD19^+^CD45^+^ blasts in the murine peripheral blood) reached approximately 35%. Mice received a single tail vein injection of 2.5 × 10^5^ TCID_50_/g body weight or a respective volume of Opti-MEM (Gibco). Two days after MV injection, mice received 1 × 10^7^ human PBMCs pooled from healthy donors or an equal volume of D-PBS (Gibco). An additional control group received human PBMCs in combination with 0.25 mg/kg commercial CD19/CD3-bsTE (endotoxin ≤18.11 EU/mg; BPS Biosciences). To assess early MV therapy efficacy for PDX #1, mice were sacrificed on day 0 (*n* = 4), day 4, and day 8 (each *n* = 4 per group). For survival analysis, PDX #1 was injected in five treatment groups (PBMC-only control, MV-Blina with PBMC, MV-Edm with PBMC, MV-Blina without PBMC, control with PBMC and CD19/CD3-bsTE; *n* = 10 mice per group) and PDX #2 in three treatment groups (PBMC-only control, MV-Blina with PBMC, MV-Edm with PBMC; each *n* = 10 mice per group). Mice were monitored for health status, body weight, and blood counts, as described above, every second day for the initial two weeks after treatment start and later once per week. Experiments were terminated at twice the duration of the final sacrifice time in the PBMC-only control group. Mice were euthanized as soon as they appeared moribund (as defined by continuous and rapid weight loss, ruffled fur, hunched posture, reduced activity, and signs of lethargy) or at the endpoint of the experiment (day 70), and necropsies were performed. Cells from bone marrow, spleen, and meninges were collected. Graft-versus-host disease (GvHD) after human PBMC transfers was ruled out at the time of death by the histopathological examination of jejunal sections, with the confirmation of apoptotic crypts via immunohistochemical staining for cleaved caspase-3. Microscopic photos were acquired using a Keyence BZ-9000E microscope and analyzed using BZ-II Viewer and BZ-II Analyzer v2.1 software (Keyence, Neu-Isenburg, Germany).

### Measles virus-Blina toxicity *in vivo*

IFNAR^−/−^ CD46 Ge mice were used to assess the toxicity of MV-Blina *in vivo*. Young (28–42 days old) and older (43–56 days old) mice were used for early time point analyses on day 0 (*n* = 4 per age and group) and day 8 (*n* = 4 per age and group) upon MV-Blina inoculation. For survival analysis, only young mice were considered (*n* = 8 CP, *n* = 10 CP + MV-Blina, *n* = 9 NaCl, *n* = 10 NaCl + MV-Blina).

Mice received two intravenous injections on day −4 and day −1 of 150 mg/kg cyclophosphamide (CP/Endoxan, Baxter, Unterschleiβheim, Germany) in a maximum of 5 mL/kg 0.9% NaCl or received an equivalent volume of 0.9% NaCl (B. Braun, Melsungen, Germany) as control. MV-Blina was injected via the tail vein on day 0 and day 7 at 1.4 × 10^10^ TCID_50_/kg body weight per dose. Mice were examined for health status, body weight, and peripheral blood counts. Blood was monitored by differential blood count (Scil Vet abc Plus+, Viernheim, Germany) and flow cytometry using anti-CD3 BV786 (564010), anti-CD4 AF647 (557681), anti-CD8 Pacific Blue (558106), and anti-CD19 PE (557399) (all 1:20 rat anti-mouse, all from BD Biosciences). Flow cytometry was performed with the Attune NxT Flow Cytometer (Thermo Fischer Scientific) and analyzed using FlowJo software (BD Biosciences). For early time points, mice were euthanized, and complete necropsies were performed on day 0 or on day 8. Mice in the survival experiment were euthanized when appearing moribund or at 100 days post treatment, and complete necropsies were performed. Serum was obtained from whole blood taken from the heart when mice were euthanized, frozen at −20°C for short-term storage, and used to assess renal and hepatic parameters at the Institute of Clinical Chemistry, Ulm University Medical Center. To assess immune cells in bone marrow, femurs were flushed with PBS, and a fraction of cells was stained for flow cytometry. Organs were kept in RNAprotect (Qiagen) and stored short-term at −20°C.

### Histology and immunohistochemistry

Formalin-fixed, paraffin-embedded tissue sections from all *in vivo* experiments were stained with hematoxylin and eosin (H&E). Immunohistochemistry for CD22 was performed using a mouse anti-human CD22 antibody (clone OTI1F2, 1:75, TA506404, Origene, Rockville, MD), for Ki67 using a mouse anti-human Ki67 antibody (clone MIB-1, 1:150, M7240, Dako, Waldbronn, Germany) and for cleaved caspase-3 (CC3) using a rabbit-anti human active caspase-3 antibody (1:200, ab13847, Abcam, Cambridge, UK). Sections were deparaffinized, pressure-cooked in citrate buffer for antigen retrieval, permeabilized with 0.1% Triton X-100 in TBS, and blocked using BLOXALL (SP-6000-100, Vector Laboratories, Newark, CA), followed by incubation with 2.5% normal horse serum (NHS, ENH-9020, Biozol) according to the manufacturer’s protocol. Sections were incubated overnight at 4°C with primary antibodies for CD22 and Ki67 diluted in 2.5% NHS. Slides were incubated with secondary antibody solution using the ImmPRESS-AP Horse anti-Mouse IgG Polymer Detection Kit (MP-5402, Vector Laboratories) or ImmPRESS HRP Horse anti-Rabbit IgG Polymer Detection Kit (MP-7401, Vector Laboratories). For substrate detection, samples were conducted with the ImmPACT Vector Red Substrate Kit (SK 5105, Vector Laboratories) or with ImmPACT DAB Peroxidase Substrate (SK-4105, Vector Laboratories) according to the manufacturer’s protocol. Sections were counterstained with Meyer’s hematoxylin (51275, Sigma-Aldrich).

For the assessment of neurotoxicity *in vitro*, cells seeded on 8-well chambers were fixed with 4% PFA (Sigma-Aldrich), permeabilized with 0.1% Tween 20 (Sigma-Aldrich) in PBS, and blocked with 10% NGS (Sigma-Aldrich) and 1% BSA (Serva) in PBS for 1 h at RT. To detect measles virus and cell structures, cells were incubated overnight at 4°C with mouse anti-MV-H antibody (1:1000, MAB8905, Merck Millipore) and rabbit-anti-alpha-Tubulin antibody (1:1000, ab52866, abcam) in 10% BSA in PBS. Secondary antibodies were applied for the detection of goat-anti-rabbit IgG (H&L) AF488 (1:300, ab150077, abcam) and goat-anti-mouse IgG1 AF594 (1:300, A21125, Invitrogen). For nuclear visualization, cells were further stained with DAPI (1:5000, Hoechst, Invitrogen).

Microscopic photos were acquired using a Keyence BZ-9000E microscope and analyzed using BZ-II Viewer and BZ-II Analyzer v2.1 software (Keyence, Neu-Isenburg, Germany).

### RNA isolation, mRNA expression, and quantitative reverse transcription polymerase chain reaction

Total RNA from cell cultures, spleen, bone marrow, and meningeal cells was isolated using the Quick-RNA Miniprep Kit (Zymo Research, Irvine, CA). Total RNA from 30 mg tissues of IFNAR^−/−^ CD46 Ge mouse tissues (spleen, brain, liver, and lung) was isolated using Lysing Matrix D tubes (MP Biomedicals, Santa Ana, CA), the TissueLyser LT, and the RNeasy Mini Kit (both Qiagen), according to the manufacturer’s protocols. RNA concentration was measured using the NanoDrop spectrophotometer (Thermo Fisher Scientific). For quantitative reverse transcription polymerase chain reaction (qRT-PCR), 500 ng RNA was reverse transcribed into complementary DNA (cDNA) using the SuperScript III First-Strand Synthesis SuperMix (Thermo Fisher Scientific). qRT-PCR was performed using the SsoAdvanced Universal SYBR Green Supermix and the CFX Connect Real-Time PCR Detection System (Bio-Rad). Relative mRNA expression was calculated as indicated in the figure legends.

For amplification, the following primers were used: secBlina forward 5′-tagcagtaccgcctatatgcagct-3′, reverse 5′-tatgaagagaaggcatacccactg-3’; MV-N forward 5′- gagaagccagggagagctacag-3′, reverse 5′- gggcagctctcgcatcac-3’; MV-P forward 5′- gtcctgtcctgggttgtctg-3′, reverse 5′-aggcacgccatgtcaaaaac-3’;

MV-L forward 5′-gtatgctcgagtccctcacg-3′, reverse 5′-gggccggataactcctaagc-3’; human β-actin forward 5′-tcaccctgaagtaccccatc-3′, reverse 5′-tagcacagcctggatagcaa-3’; human GAPDH forward 5′-gacaactttggtatcgtggaa-3′, reverse 5′-ccaggaaatgagcttgaca-3’; human Nestin forward 5′-tcaagatgtccctcagcctgga-3′, reverse 5′-aagctgagggaagtcttggagc-3’; human PHOX2B forward 5′-cctgaagatcgacctcacagag-3′, reverse 5′-ttttgcccgaggagccgttctt-3’; mouse Actin b forward 5′-cactgtcgagtcgcgtcc-3′, reverse 5′-tcatccatggcgaactggtg-3’; mouse Gusb forward 5′-cgctacgggagtcgggc-3′, reverse 5′-ggtatcttgggtccatcgcc-3’.

### Measles-specific total antibody detection

Total MV-specific IgG levels in the serum of infected IFNAR^−/−^ CD46 Ge mice were determined by an enzyme-linked immunosorbent assay (ELISA). 96-well NUNC MaxiSorp plates (Thermo Fisher Scientific) were coated overnight at 4°C with 10 μg/ml MV bulk antigens (Serion Immunologics, Würzburg, Germany). An anti-MV-N antibody (MAB8906, Merck Millipore) was used as a standard. Wells were blocked with 5% BSA (Serva) and 0.1% Tween 20 (Sigma-Aldrich) for 2 h at RT. Standard and serum samples of infected mice were applied in serial dilutions in duplicates and incubated for 2 h at RT. For detection, samples were incubated with a secondary goat anti-mouse IgG (H + L) HRP antibody (Bio-Rad) for 1 h at RT. The OptEIA TMB Substrate Reagent (BD Biosciences) was applied according to the manufacturer’s protocol and stopped using 1.75 N sulfuric acid (Sigma-Aldrich). Absorbance was measured at 450 nm and 650 nm reference using the Infinite M200 microplate reader (Tecan).

### Statistical analysis

Statistical analyses were performed using GraphPad Prism 8.4.3 software (GraphPad Software, San Diego, CA). Data are presented as mean ± SD. Three independent experiments were performed, unless otherwise noted. Appropriate sample sizes for animal experiments were determined with the assistance of a biometrician. Comparisons between two groups were performed using two-tailed unpaired Student’s t-tests. Comparison between more than two groups was performed using ANOVA with a multiple comparison test. Kaplan-Meier curves were generated for animal survival, and statistical analysis was assessed using the log rank test (Mantel-Cox). Results were considered significant if *p* < 0.05.

## Data availability

All data generated or analyzed during this study are included in the main text or supplemental information. Further enquiries should be addressed to the corresponding author (C.B.).

## Acknowledgments

We extend our sincere gratitude to Irmela Jeremias for generously providing the PDX samples ALL-199 and ALL-265. We also deeply appreciate the outstanding technical assistance of Helgard Knauss. This work was supported by the German National Science Foundation (DFG BE 2416/12-1 granted to C. Beltinger), as well as the Start-Up Funding Program of the Medical Faculty (Bausteinprogramm) granted to C. Dorneburg. We gratefully acknowledge Hartmut Geiger, head of the Institute of Molecular Medicine, and Karina Eiwen for providing access to the scil Vet for mouse blood measurements. We thank the Central Facility (ZE) for Clinical Chemistry at the Ulm University Medical Center. S. Heinze and G. L. Stadler were members of the International Graduate School in Molecular Medicine in Ulm, Germany. The Graphical Abstract and Figures 1A, 4A, 6A, and 7A were created using Biorender.com.

## Author contributions

C.B., C.D., and S.H. conceptualized the project. S.H., C.D., and T.F.E.B. developed the methodology. S.H. conducted the investigation and curated the data. S.H. and C.B. wrote the original article draft. C.B., S.H., C.E.E., C.D., M.E., and K.M.D. reviewed and edited the article. G.L.S., J.P.W.H., C.E.E., L.H.M., and M.D.M. provided resources and technical or material support. C.B. supervised the project. C.B. and C.D. acquired funding.

## Declaration of interests

The authors declare no competing interests.

## Supplemental information

Document S1. Figures S1–S13

Document S2. Article plus supplemental information
