# Supplementary material for: A measles virus encoding a CD19/CD3 bispecific T cell engager shows enhanced preclinical anti-BCP-ALL efficacy without significant toxicity
Source: Mol Ther Oncol. 2026 Jan 10;34(1):201127. doi: 10.1016/j.omton.2026.201127 (PMC12860616; doi:10.1016/j.omton.2026.201127)
Supplement: Document S1. Figures S1–S13 [file mmc1.pdf]

OMTON, Volume *34*

Supplemental information

A measles virus encoding a CD19/CD3 bispecific T cell engager shows enhanced preclinical anti-BCP-ALL efficacy without significant toxicity

Sabine Heinze, Giovanna L. Stadler, Yonghui Zhang, Christine E. Engeland, Thomas F.E. Barth, Johannes P.W. Heidbuechel, Lüder H. Meyer, Michael D. Mühlebach, Klaus-Michael Debatin, Carmen Dorneburg, and Christian Beltinger

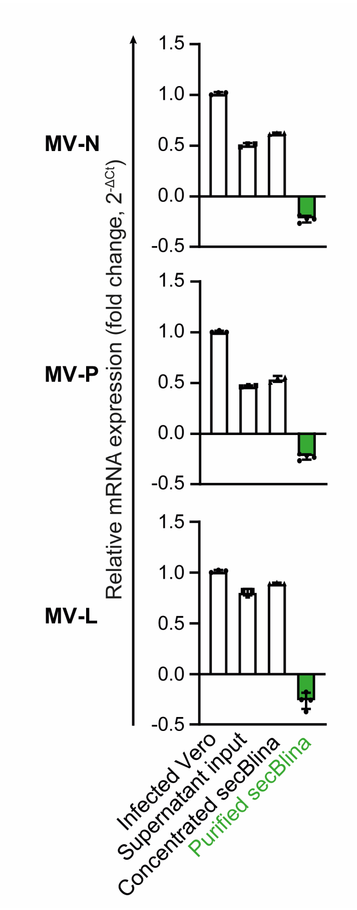

Figure S1. secBlina is devoid of MV particles. Purified and concentrated secBlina, harvested from infected Vero cell supernatant, was compared to infected Vero cells, supernatant input and concentrated supernatant before secBlina purification. Expression of mRNA of the MV genome (MV-N, MV-P and MV-L) was determined by qRT-PCR, calculated by $2^{-\Delta Ct}$ method and normalized to infected Vero cells to show relative expression. Results are shown as means ± SD of n = 3 independent experiments.

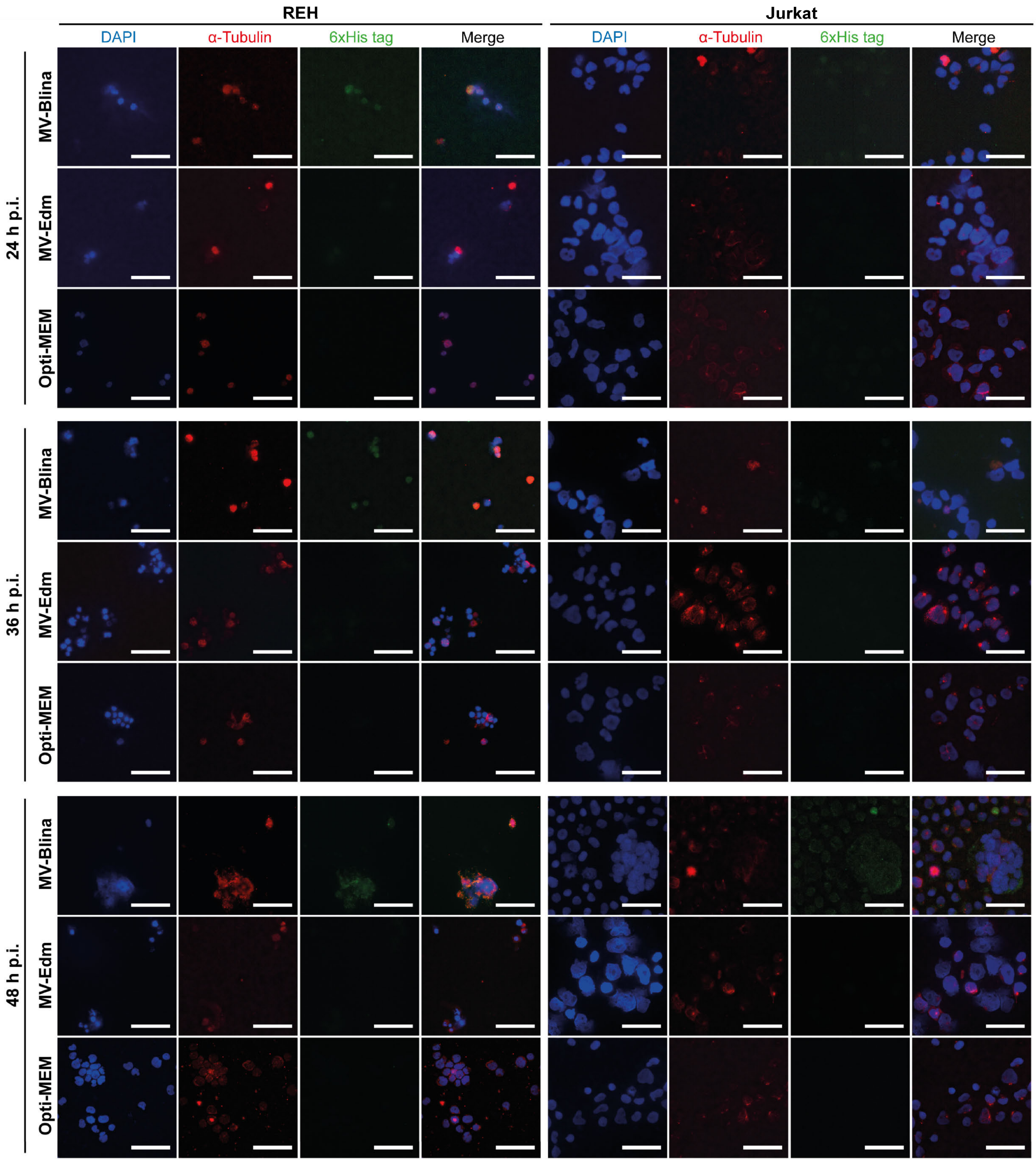

Figure S2. MV-Blina infected leukemia cells produce and secrete secBlina at early time points. REH and Jurkat cells are shown for 24 hours, 36 hours and 48 hours post MV infection or Opti-MEM control. secBlina was detected by His-tag IF staining. Scale bare represent 50 µm. Representatives of three independent experiments are shown.

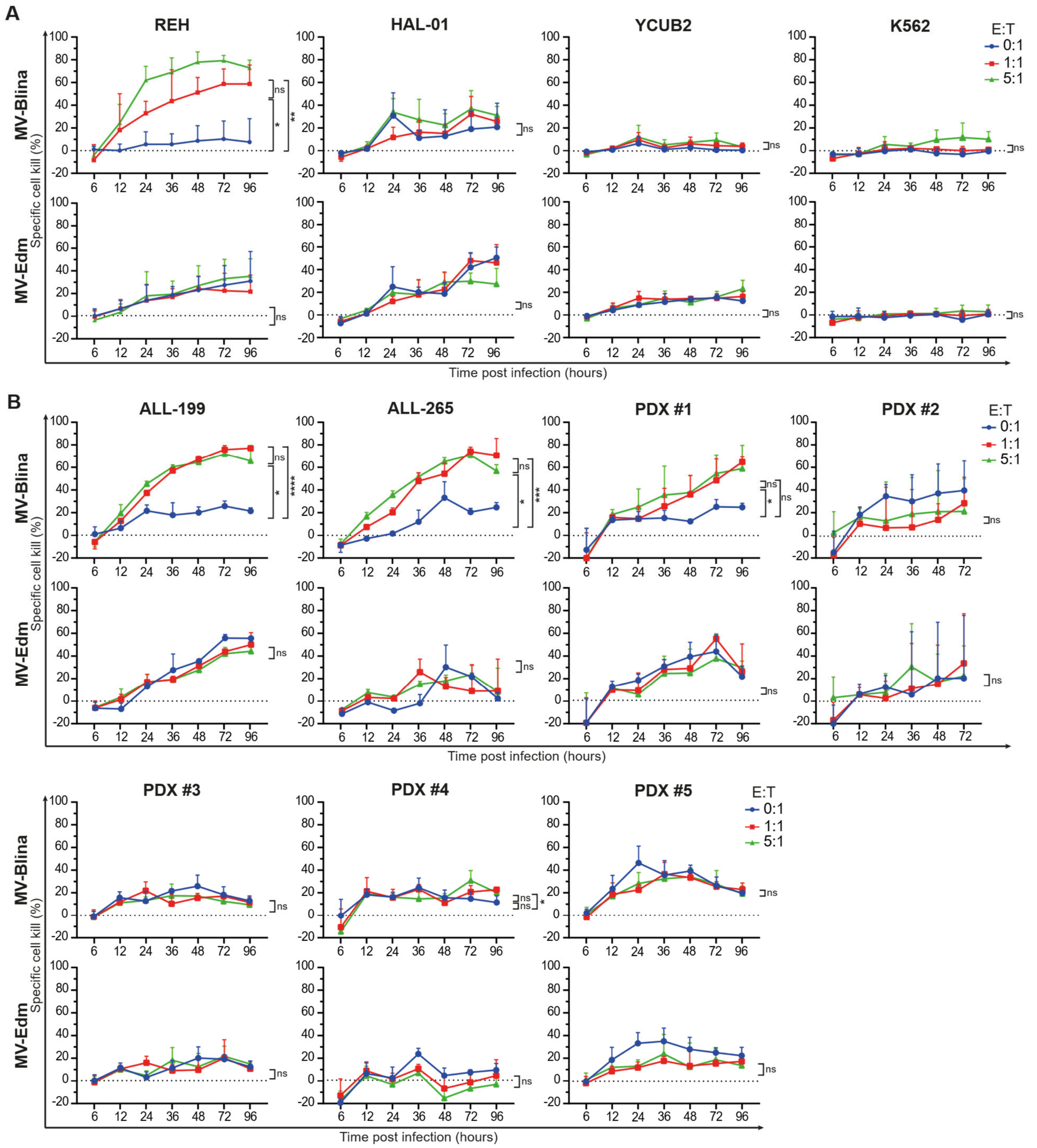

Figure S3. ALL cell killing mediated by secBlina in the presence of PBMCs upon MV infection with MOI of 1.0. A) More prominent, albeit heterogenous, killing of target ALL cell lines upon MV-Blina treatment. Target cell lines were infected with MV at MOI 1.0 and cocultured with pooled PBMCs of healthy donors at different E:T ratios. Specific cell kill was measured by flow cytometry at the time indicated. Results are shown as mean ± SD of n = 5 independent experiments. **B) secBlina enhances killing of some PDX independent of their risk at a higher MOI of 1.0.** Target PDX cells were infected with MV and co-cultured with pooled PBMCs of healthy donors at different E:T ratios. Specific cell kill was measured by flow cytometry at the time indicated. Results are shown as mean ± SD of n = 4 independent experiments. Statistical analysis was performed using two-way ANOVA with Tukey's correction. ns, not significant; *, $p < 0.05$; **, $p < 0.01$; ***, $p < 0.001$; ****, $p < 0.0001$. T, target cells; E, effector cells; PDX, patient-derived xenografts.

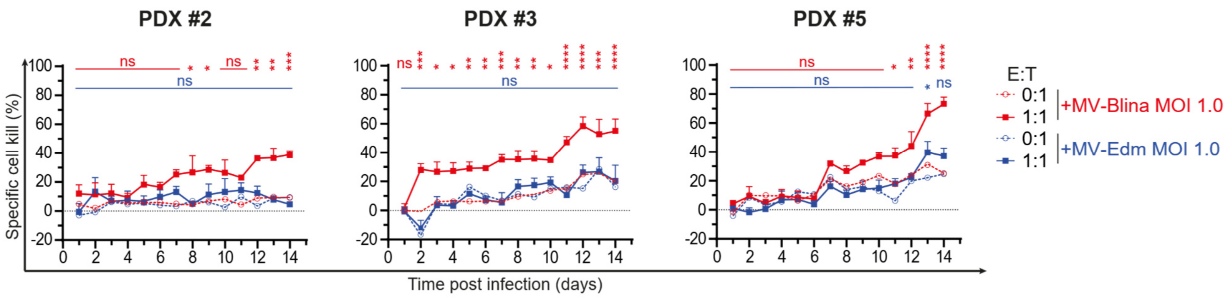

Figure S4. Additive secBlina-mediated effect in PDX cell kill using OP9 feeder cells. Target (T) PDX cells were infected with MV and cocultured with pooled PBMCs of healthy donors (E, effector cells) at an E:T ratio of 1:1 or 0:1 in the presence of OP9 feeder cells. Specific cell kill was measured by flow cytometry at the time indicated. Results are shown as mean ± SD of n = 4 independent experiments for 1:1 ratio or n = 1 for 0:1 ratio. Statistical analysis was performed using two-way ANOVA with Tukey's correction. ns, not significant; *, $p < 0.05$; **, $p < 0.01$; ***, $p < 0.001$; ****, $p < 0.0001$. T, target cells; E, effector cells; PDX, patient-derived xenografts.

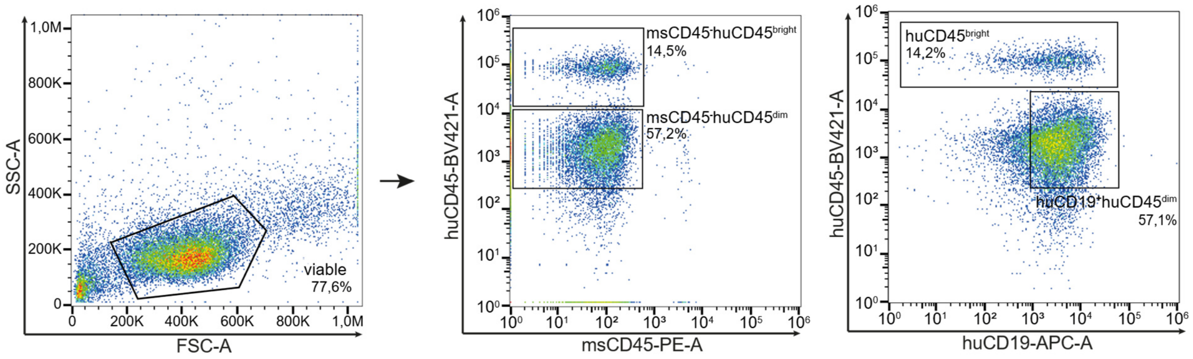

Figure S5. Gating strategy for human PDX and PBMC in mouse blood. Viable cells were selected by FSC/SSC. To distinguish between human PDX and PBMC populations, cells were defined as $msCD45^{-}$ and seperated into $huCD45^{dim}$ (PDX) and $huCD45^{bright}$ (PBMC). Analyzed percentage was validated by $huCD19^{+}$ population. Applied gates and corresponding cell populations (as % of total cells) are shown. Plots were generated using FlowJo software.

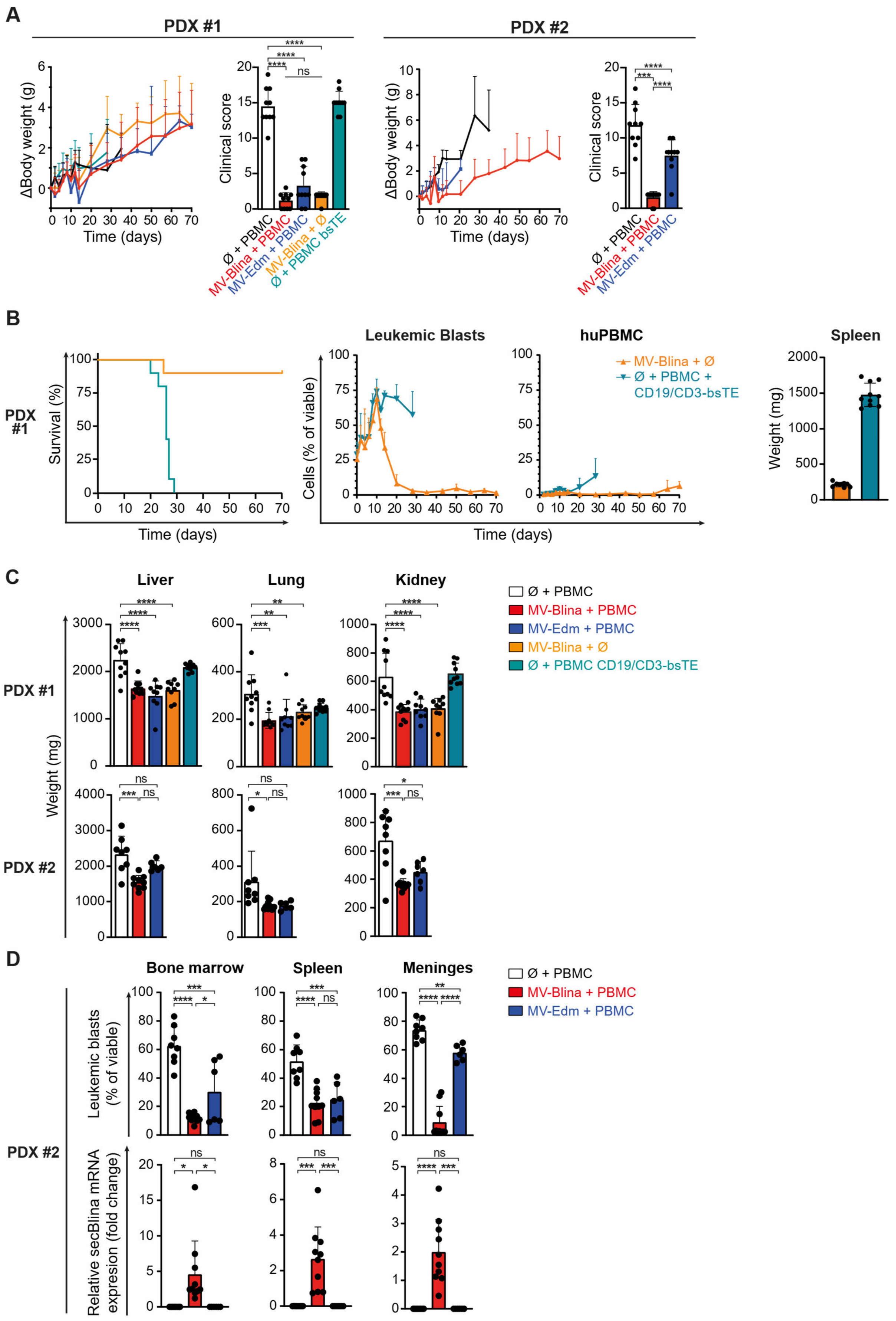
A
PDX #1
PDX #2
ΔBody weight (g)
Time (days)
Clinical score
Ø + PBMC
MV-Blina + PBMC
MV-Edm + PBMC
MV-Blina + Ø
Ø + PBMC bsTE
B
PDX #1
Survival (%)
Leukemic Blasts
huPBMC
Cells (% of viable)
MV-Blina + Ø
Ø + PBMC + CD19/CD3-bsTE
Spleen
Weight (mg)
C
Liver
Lung
Kidney
Ø + PBMC
MV-Blina + PBMC
MV-Edm + PBMC
MV-Blina + Ø
Ø + PBMC CD19/CD3-bsTE
D
Bone marrow
Spleen
Meninges
PDX #2
Leukemic blasts (% of viable)
Relative secBlina mRNA expresion (fold change)

Figure S6. Significant improvement of health status, disease-related stress and leukemic load. A) Significantly better clinical outcome with MV treatment with or without PBMCs. Mice were assessed for general health status, body weight and disease-related stress to determine the clinical score (continuous and rapid weight loss, ruffled fur, hunched posture, reduced activity and signs of lethargy). Body weight changes and the summary of clinical score for PDX #1 and #2 are shown (n = 10 per PDX and treatment groups). **B) MV-Blina reduces the leukemic burden in the absence of PBMCs.** Survival for control groups treated with MV-Blina and PBS or with Opti-MEM and a combination of CD19/CD3-bsTE with PBMCs are shown. Leukemic blasts ($msCD45^{-}huCD19^{+}huCD45^{dim}$) and human PBMC ($msCD45^{-}huCD19^{+}huCD45^{bright}$) in peripheral blood were monitored by flow cytometry at indicated time points. Spleen weight was measured at time of death. **C) Significant weight reduction of liver, lung and kidney for MV-Blina-treated PDX #1 and #2.** Weight of organs was measured at time of death. **D) In PDX #2 MV-Blina significantly reduces the number of leukemic blasts in ALL compartments.** At the time of death, leukemic blasts ($Ly5^{-}CD19^{+}CD45^{dim}$) were detected by flow cytometry in ALL compartments (bone marrow, spleen and meninges). Replication of secBlina was detected using qRT-PCR and calculated by the $2^{-\Delta\Delta Ct}$ method displaying the fold change relative to huActin and huGAPDH. At time of death, complete necropsy was performed and organs analyzed in mice with PDX #1 (n = 10 per group) and PDX #2 (MV-Blina with PBMC, n = 10; MV-Edm and PBMC, n = 6; control PBMC only, n = 8) in B-D. Statistical analysis was performed using Mantel-Cox log-rank test (B), one-way ANOVA with Tukey's multiple comparisons test(A, C, D). ns, not significant; *, $p < 0.05$; **, $p < 0.01$; ***, $p < 0.001$; ****, $p < 0.0001$.

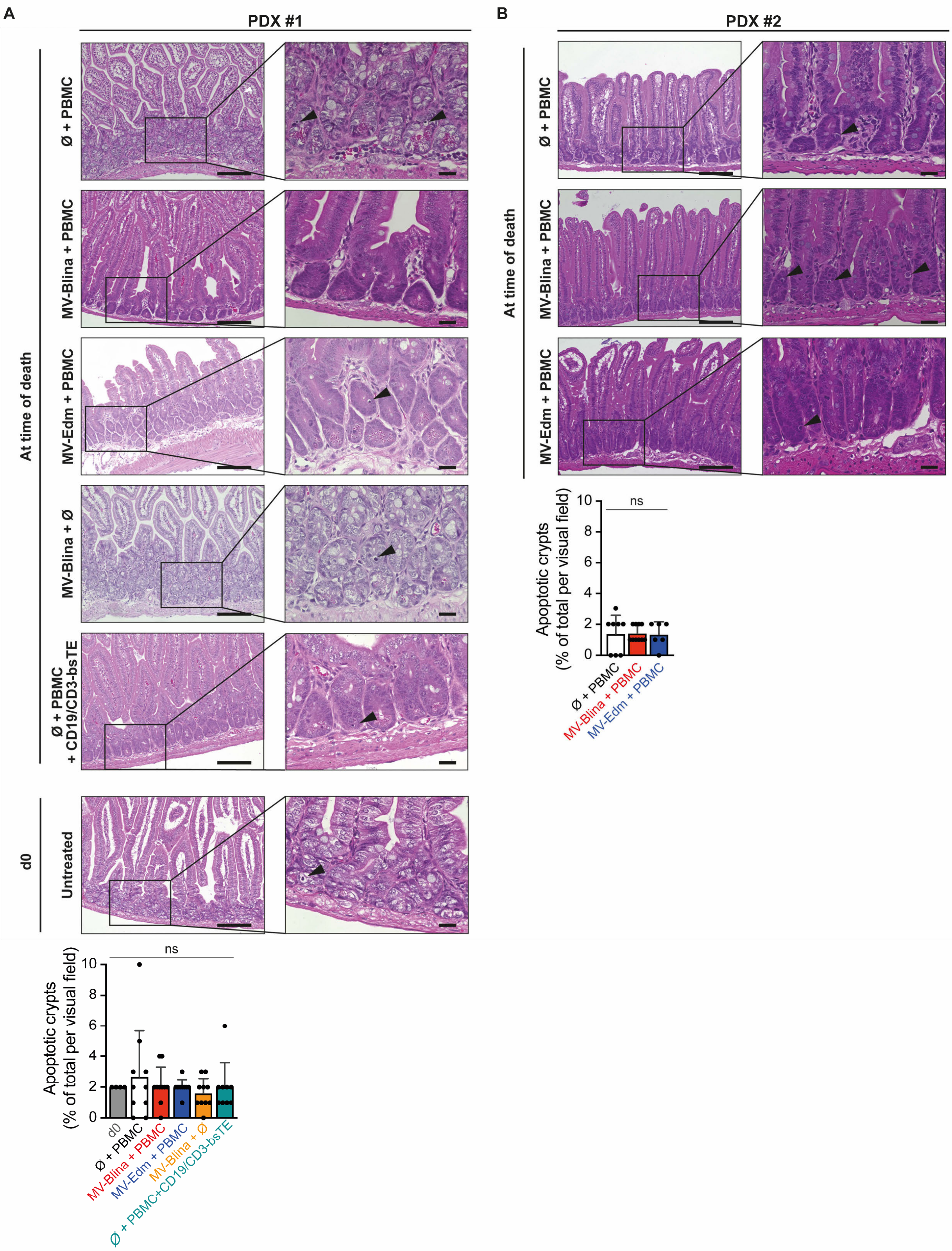
A
PDX #1
At time of death
Ø + PBMC
MV-Blina + PBMC
MV-Edm + PBMC
MV-Blina + Ø
Ø + PBMC + CD19/CD3-bsTE
d0
Untreated
Apoptotic crypts (% of total per visual field)
ns
d0
Ø + PBMC
MV-Blina + PBMC
MV-Edm + PBMC
MV-Blina + Ø
Ø + PBMC+CD19/CD3-bsTE
B
PDX #2
At time of death
Ø + PBMC
MV-Blina + PBMC
MV-Edm + PBMC
Apoptotic crypts (% of total per visual field)
ns
Ø + PBMC
MV-Blina + PBMC
MV-Edm + PBMC

Figure S7. Histopathological analysis excludes graft-versus-host disease in ALL-bearing mice. Formalin-fixed, paraffin-embedded sections of jejunal tissues were stained with H&E. For each PDX, left panels show 20x magnification (scale bar 100 µm), right panels show 60x magnification (scale bar 50 µm). Apoptotic crypts (black arrow heads) are presented as percentage of total counted crypts per visual field. **A) Jejunal tissues of PDX #1** of untreated mice at therapy start (d0, n = 4) and of therapy (control PBMC only, n = 10; MV-Blina with PBMC, n = 10; MV-Edm with PBMC, n = 9; MV-Blina without PBMC, n = 10; control with PBMC and CD19/CD3-BiTE, n = 9) were analyzed. **B) Jejunal tissues of PDX #2** of treated mice (control PBMC only, n = 8; MV-Blina and PBMC, n = 10; MV-Edm and PBMC, n = 6) were analyzed. Statistical analysis was performed using one-way ANOVA with Tukey's multiple comparisons test. ns, not significant.

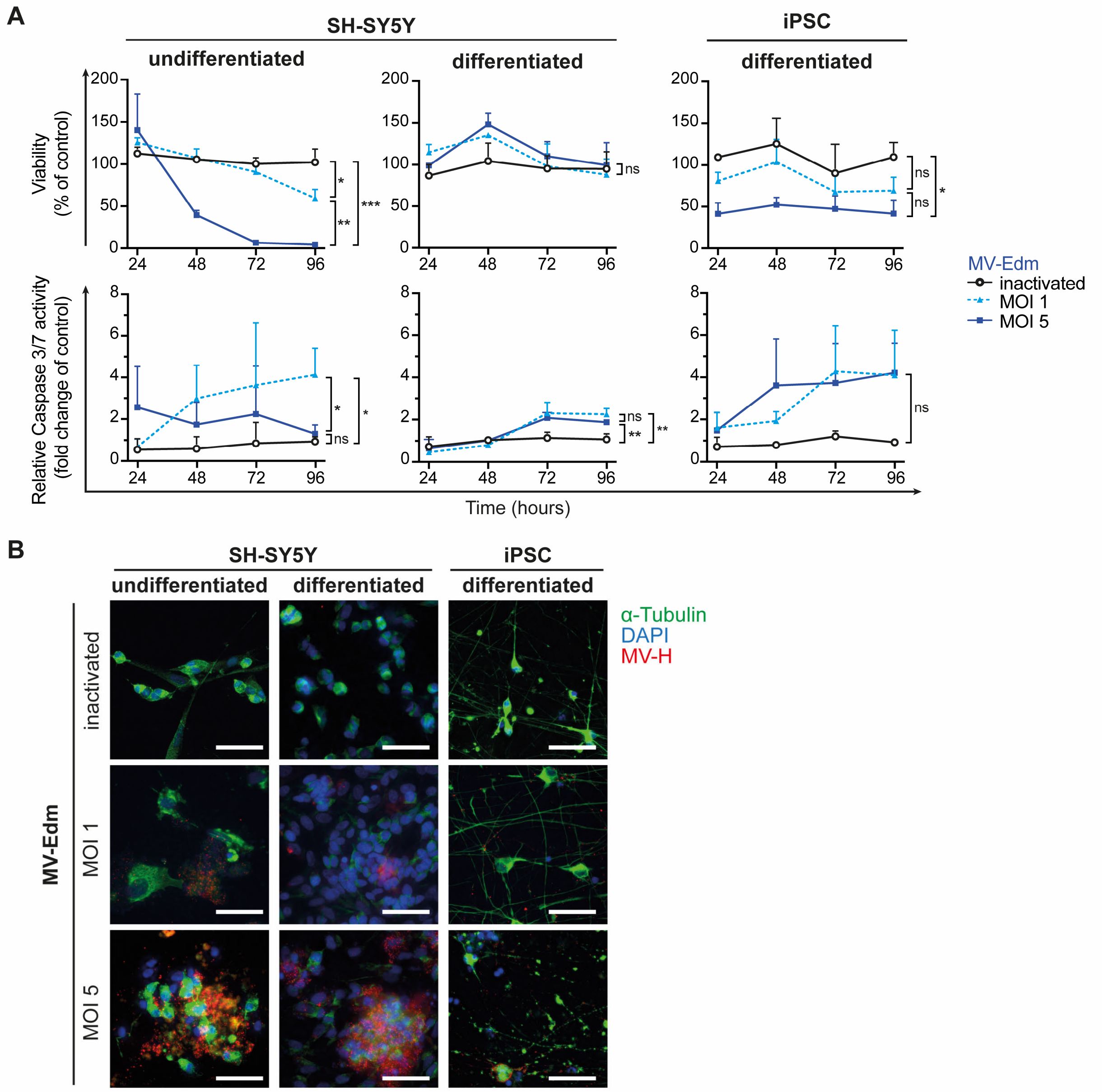

Figure S8. Mild toxicity of MV-Edm in neuronal cells in vitro. SH-SY5Y cells and human iPSC-derived neurons were tested for in vitro toxicity of MV-Edm as shown in Figure 5. **A**) **MV-Edm reduces cell viability and increases apoptosis at MOI 1 in undifferentiated SY5Y as well as in iPSC-derived neurons.** Undifferentiated and neuronally differentiated SY5Y and differentiated iPSC neurons were inoculated with either Opti-MEM (untreated control) or inactivated MV-Edm or MOI of 1 or 5 for different time periods as indicated. Viability and apoptosis, both relative to untreated control, were measured by CellTiter-Glo® assay and Caspase-Glo® 3/7 assay, respectively. Results are shown as means ± SD of n = 4 (undifferentiated and differentiated SY5Y) or n = 3 (differentiated iPSC) independent experiments. **B) Cell death and syncytium formation of differentiated neuronal cells upon MV-Edm infection.** Undifferentiated and differentiated SY5Y and differentiated iPSC neuronal cells were inoculated with either inactivated MV-Edm or MOI of 1 or 5 and stained after 96 hours. Cells were stained for α-Tubulin (green), DAPI (blue) and MV-H (red). Results are representative of n = 4 (SY5Y) or m=3 (iPSC) independent experiments. Scale bar 50 µm. Statistical analysis was performed using two-way ANOVA with Tukey's multiple comparisons test. ns, not significant; *, $p < 0.05$; **, $p < 0.01$; ***, $p < 0.001$.

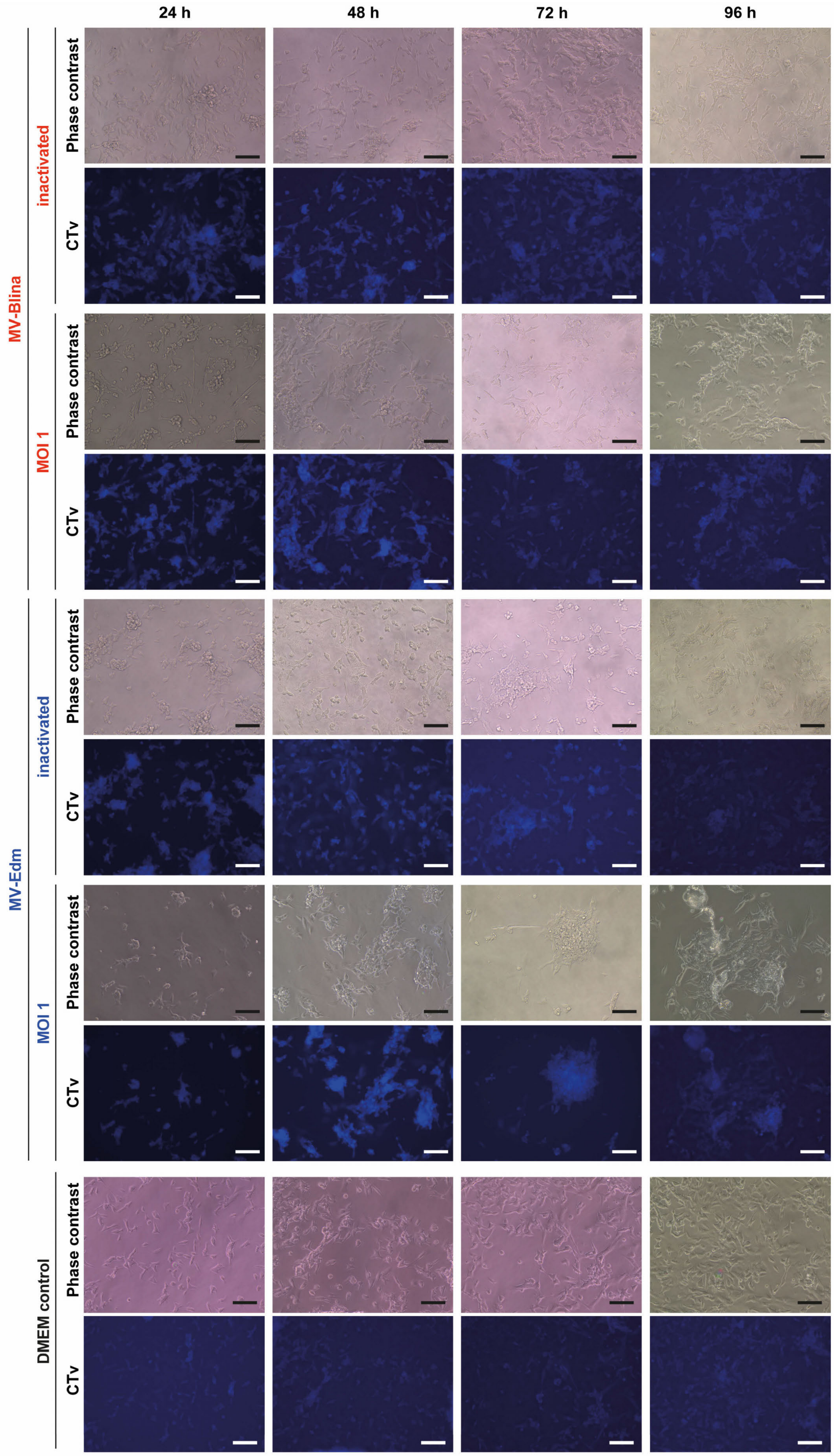
24 h
48 h
72 h
96 h
MV-Blina
inactivated
MOI 1
MV-Edm
inactivated
MOI 1
DMEM control
Phase contrast
CTv

Figure S9. No neurotoxicity in vitro of MV-Blina in differentiated SH-SY5Y cells. CTv pre-stained SH-SY5Y cells were seeded on poly-D-lysine and differentiated in DMEM-based medium containing 5 µM ATRA and 5% FBS for 11 days. On day 12, SH-SY5Y were inoculated with inactivated MV-Blina (upper panels) or MV-Edm (middle panels) or at an MOI of 1.0 with RPMI 1640-based medium. For untreated control, differentiated SH-SY5Y were maintained in DMEM-based medium. Differentiated SH-SY5Y were further cocultured for indicated time points. Cytotoxic and cytopathic effects were observed by microscopy. Representatives of n = 5 are shown. Scale bar represents 100 µm. CTv, CellTrace Violet; E, effector cells; T, target cells.

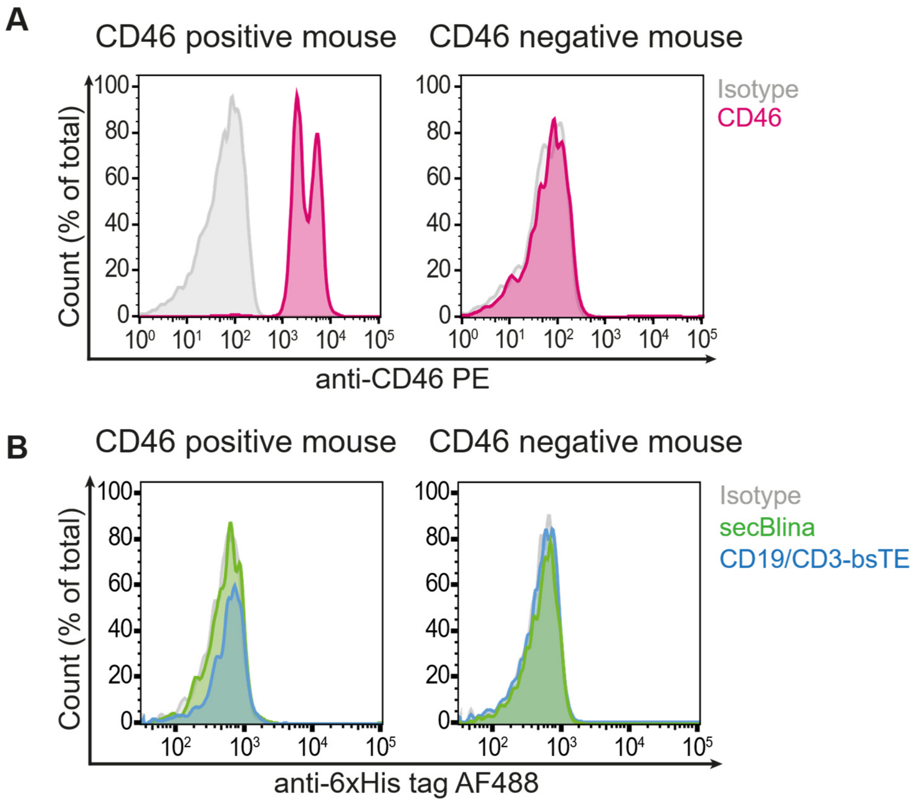

Figure S10. IFNAR$^{-/-}$ mouse peripheral blood cells are transgenic for CD46 and do not bind secBlina. A) Transgenic mice express CD46 on blood cells. Peripheral blood was taken from genotyped mice. Blood cells were stained for human CD46. **B) No unspecific binding of secBlina on murine cells.** Peripheral blood samples were incubated with either 1 µg secBlina or 1 µg commercial CD19/CD3-BiTE. Binding was validated using an anti-6x His tag antibody.

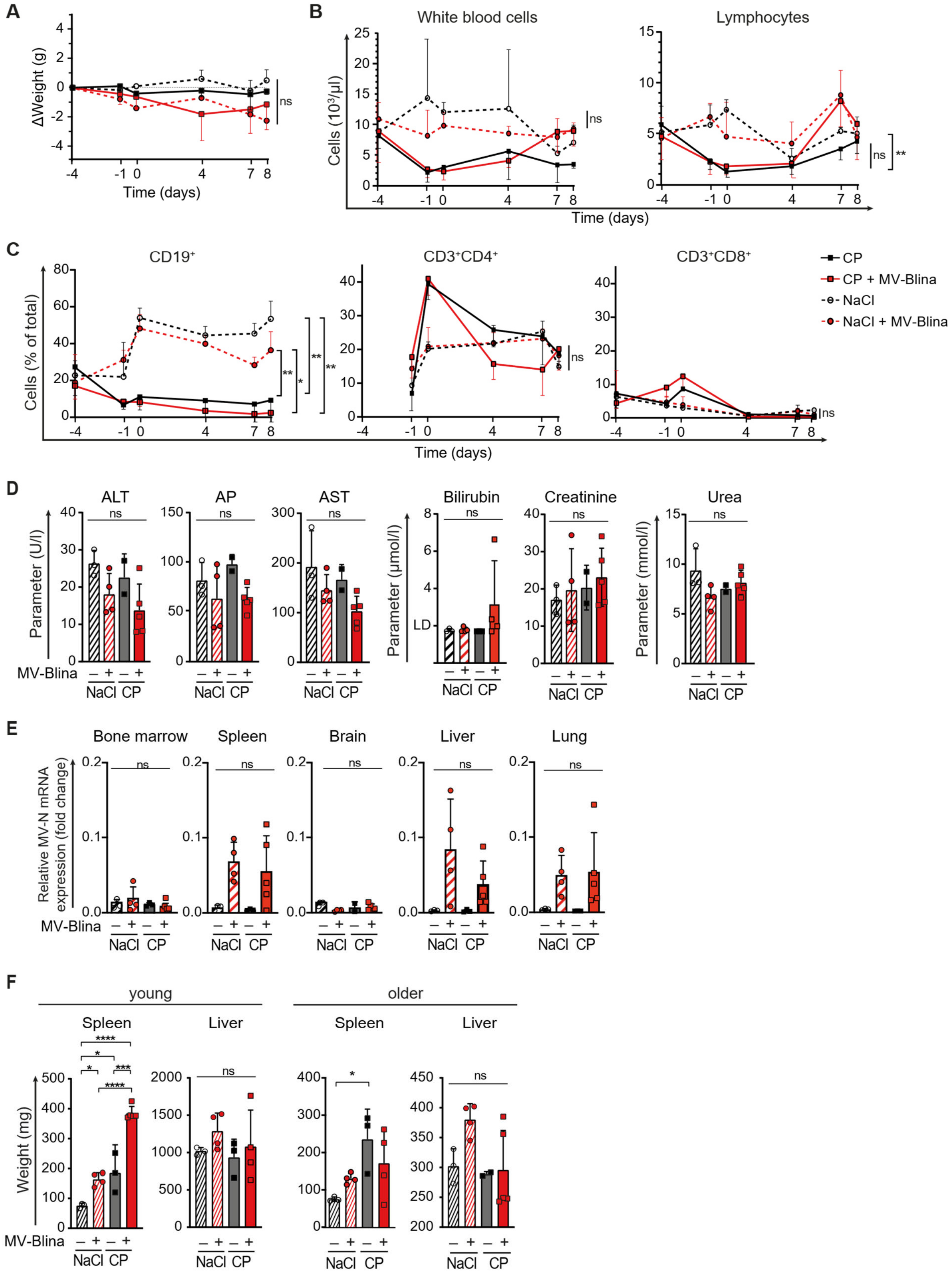
A
ΔWeight (g)
Time (days)
ns
B
White blood cells
Lymphocytes
Cells (10³/µl)
Time (days)
ns
**
C
CD19⁺
CD3⁺CD4⁺
CD3⁺CD8⁺
Cells (% of total)
Time (days)
CP
CP + MV-Blina
NaCl
NaCl + MV-Blina
D
ALT
AP
AST
Bilirubin
Creatinine
Urea
Parameter (U/l)
Parameter (µmol/l)
Parameter (mmol/l)
LD
MV-Blina
NaCl CP
E
Bone marrow
Spleen
Brain
Liver
Lung
Relative MV-N mRNA expression (fold change)
MV-Blina
NaCl CP
F
young
older
Spleen
Liver
Weight (mg)
MV-Blina
NaCl CP

Figure S11. No acute toxicity of MV-Blina in vivo. Older (43-56 days old) IFNAR$^{-/-}$CD46tg mice were injected i.v. with cyclophosphamide (CP, 150 mg/kg, solid lines) or control (0.9% NaCl, dashed lines) at day -4 and day -1. MV-Blina (1.4 x10^{10} $TCID_{50}$/kg, red) or control (PBS, black) was administered twice on day 0 and day 7. Mice were sacrificed on day 8 (CP, n = 3; CP with MV, n = 5; NaCl, n = 3; NaCl with MV-Blina, n = 4). **A) No weight loss**. **B) B-cell depletion after two i.v. injections of CP.** White blood cells and lymphocytes of peripheral blood were assessed. Percentages of msCD19$^+$ B cells, msCD3$^+$CD4$^+$ and msCD3$^+$CD8$^+$ T-cells in peripheral blood were determined by flow cytometry. **D) No hepatic or renal short-term toxicity.** Serum hepatic parameters (ALT, AST, AP, bilirubin) and renal parameters (creatinine, urea) were evaluated on day 8. **E) MV-Blina infects liver, lung and spleen but not bone marrow and brain.** RNA was isolated from indicated tissues at time of sacrifice. Expression of measles virus mRNA (MV-N) was detected using qRT-PCR and fold change was calculated by the $2^{-\Delta\Delta Ct}$ method relative to msActin and msGusb. **F) CP redirects hematopoiesis in older mice toward extramedullary sites.** Spleen and liver weights were assessed on day 8. All results are shown as means ± SD. Statistical analysis was performed using two-way ANOVA with Tukey's multiple comparisons test (A-C) and one-way ANOVA with Tukey's comparisons test (D-F). ns, not significant; *, $p < 0.05$; **, $p < 0.01$; ***, $p < 0.001$; ****, $p < 0.0001$.

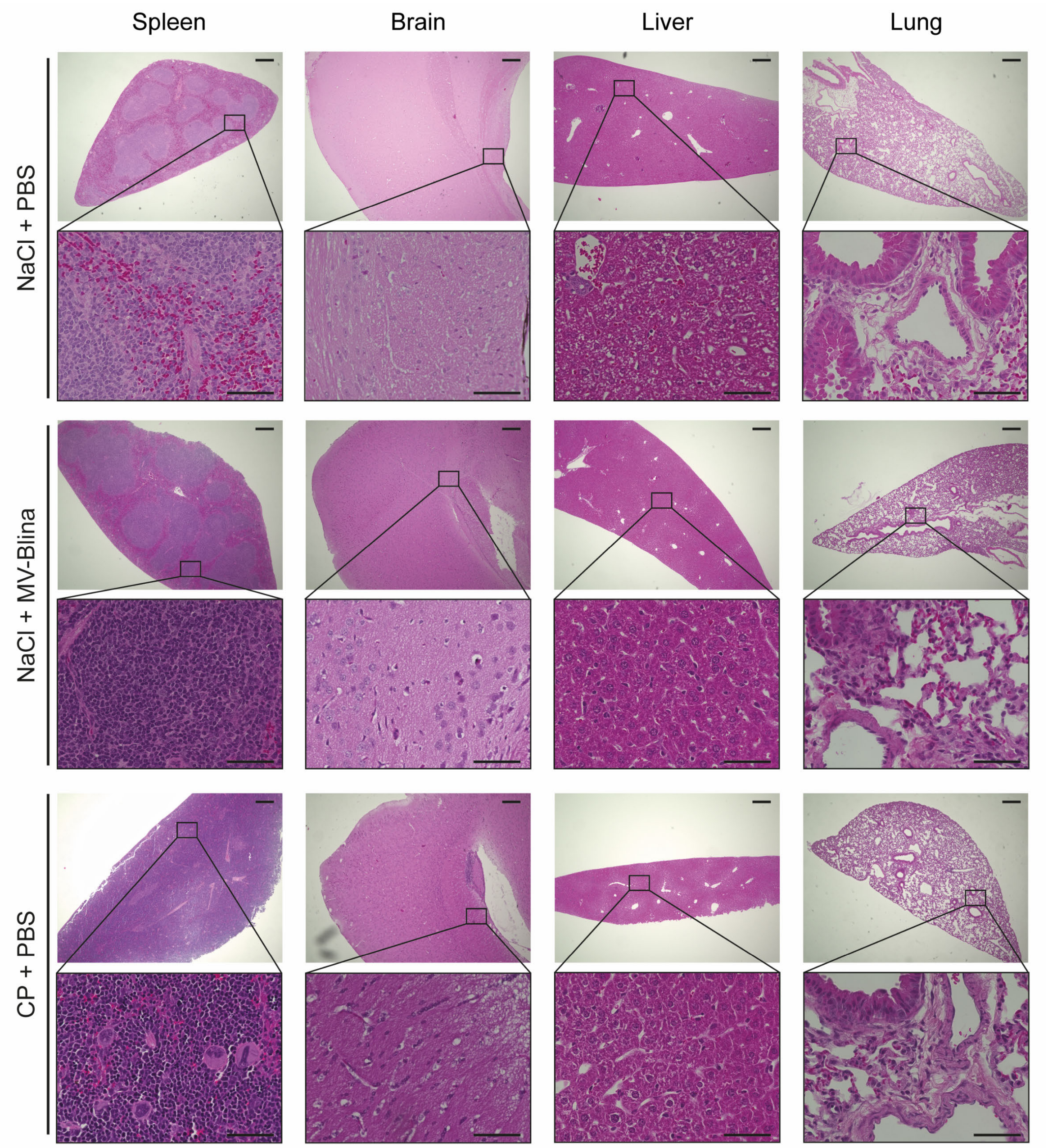

Figure S12. No histological signs of toxicity in spleen, brain, liver and lung in young mice. Young IFNAR$^{-/-}$ CD46 Ge mice were treated as described before. Tissue sections of indicated organs were stained with H&E to assess histological signs of toxicity. Representative sections are shown for mice subjected to NaCl control, NaCl with MV-Blina or CP control. Scale bars represent 300 μm (upper panels) and 50 μm (lower panels).

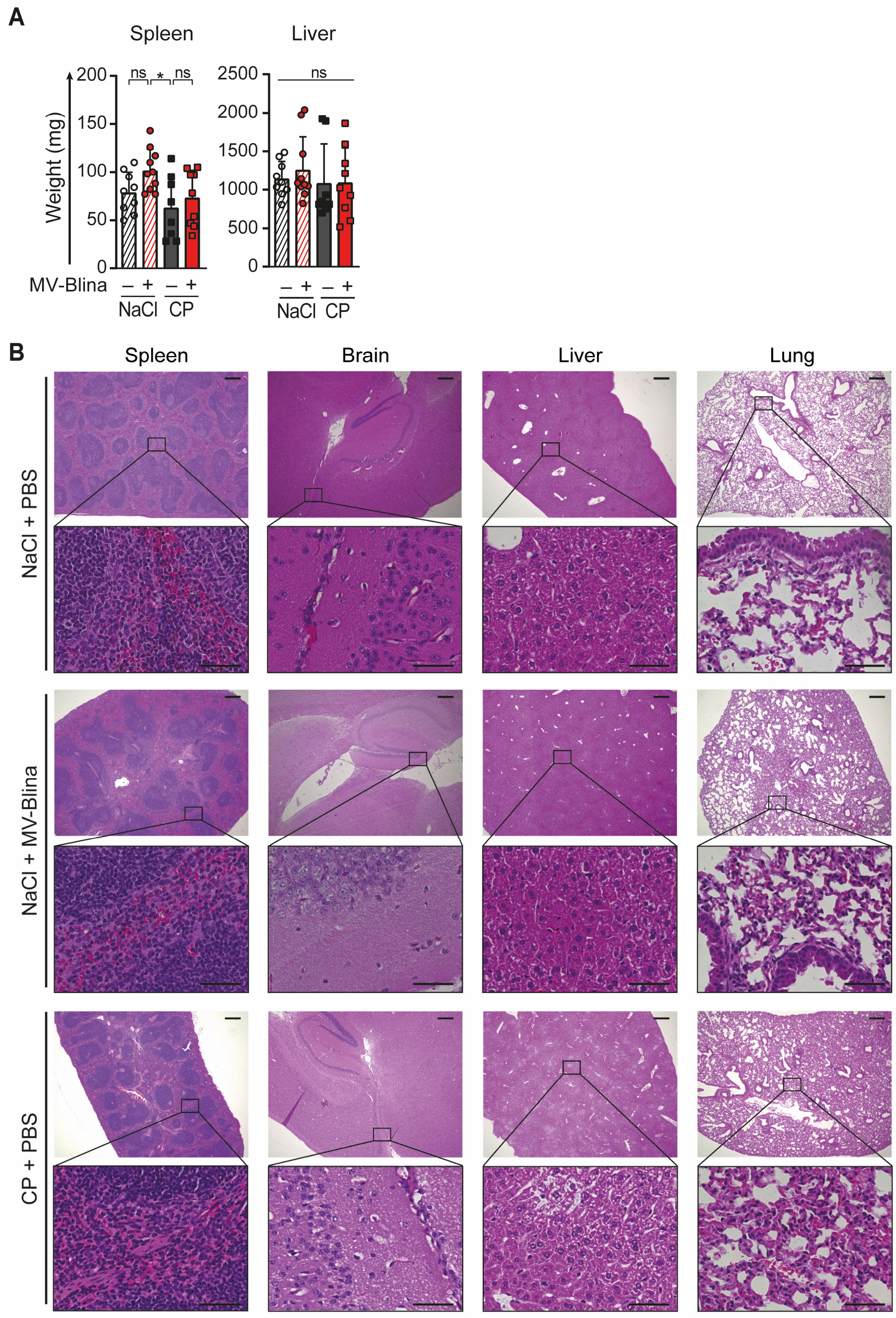
A
Spleen
Liver
Weight (mg)
200
150
100
50
0
ns
*
ns
2500
2000
1500
1000
500
0
MV-Blina
– + – +
NaCl CP
B
Spleen
Brain
Liver
Lung
NaCl + PBS
NaCl + MV-Blina
CP + PBS

Figure S13. No long-term toxicity in young MV-Blina-treated mice. Young IFNAR$^{-/-}$ CD46 Ge mice were treated as described before to assess MV-Blina toxicity in vivo. (CP, n = 8; CP with MV, n = 10; NaCl, n = 9; NaCl with MV-Blina, n = 10). **A) Treatment with CP and MV-Bina does not cause long-term damage.** Weight of spleen and liver was determined at time of death. All results are shown as means ± SD. Statistical analysis was performed using one-way ANOVA with Tukey's comparisons test. ns, not significant; *, $p < 0.05$. **B) No histological signs of long-term toxicity.** Tissue sections of indicated organs were stained with H&E to assess histological evidence of toxicity. Representative sections are shown for mice subjected to NaCl control, NaCl with MV-Blina or CP control. Scale bars represent 300 µm (upper panels) and 50 µm (lower panels).
